# Effects of fructose-containing sweeteners on fructose intestinal, hepatic, and oral bioavailability in dual-catheterized rats

**DOI:** 10.1371/journal.pone.0207024

**Published:** 2018-11-08

**Authors:** Leah R. Villegas, Christopher J. Rivard, Brandi Hunter, Zhiying You, Carlos Roncal, Melanie S. Joy, MyPhuong T. Le

**Affiliations:** 1 Cardiovascular Pulmonary Research Laboratories, Departments of Pediatrics and Medicine, University of Colorado Anschutz Medical Campus, Aurora, Colorado, United States of America; 2 Division of Renal Diseases and Hypertension, Department of Medicine, University of Colorado Anschutz Medical Campus, Aurora, Colorado, United States of America; 3 Department of Pharmaceutical Sciences, Skaggs School of Pharmacy, University of Colorado Anschutz Medical Campus, Aurora, Colorado, United States of America; Universidade do Estado do Rio de Janeiro, BRAZIL

## Abstract

**Objective:**

Fructose is commonplace in Western diets and is consumed primarily through added sugars as sucrose or high fructose corn syrup. High consumption of fructose has been linked to the development of metabolic disorders, such as cardiovascular diseases. The majority of the harmful effects of fructose can be traced to its uncontrolled and rapid metabolism, primarily within the liver. It has been speculated that the formulation of fructose-containing sweeteners can have varying impacts on its adverse effects. Unfortunately, there is limited data supporting this hypothesis. The objective of this study was to examine the impact of different fructose-containing sweeteners on the intestinal, hepatic, and oral bioavailability of fructose.

**Methods:**

Portal and femoral vein catheters were surgically implanted in male Wistar rats. Animals were gavaged with a 1 g/kg carbohydrate solution consisting of fructose, 45% glucose/55% fructose, sucrose, glucose, or water. Blood samples were then collected from the portal and systemic circulation. Fructose levels were measured and pharmacokinetic parameters were calculated.

**Results:**

Compared to animals that were gavaged with 45% glucose/55% fructose or sucrose, fructose-gavaged animals had a 40% greater fructose area under the curve and a 15% greater change in maximum fructose concentration in the portal circulation. In the systemic circulation of fructose-gavaged animals, the fructose area under the curve was 17% and 24% higher and the change in the maximum fructose concentration was 15% and 30% higher than the animals that received 45% glucose/55% fructose or sucrose, respectively. After the oral administration of fructose, 45% glucose/55% fructose, and sucrose, the bioavailability of fructose was as follows: intestinal availability was 0.62, 0.53 and 0.57; hepatic availability was 0.33, 0.45 and 0.45; and oral bioavailability was 0.19, 0.23 and 0.24, respectively.

**Conclusions:**

Our studies show that the co-ingestion of glucose did not enhance fructose absorption, rather, it decreased fructose metabolism in the liver. The intestinal, hepatic, and oral bioavailability of fructose was similar between 45% glucose/55% fructose and sucrose.

## Introduction

Fructose is pervasive throughout Western diets. Since the 1960s, fructose consumption has risen sharply.[1] Currently, individual consumption is estimated to be 50 to 70 g of fructose daily, accounting for 10 to 15% of the total dietary caloric intake.[2, 3] This high consumption of fructose presents a serious public health concern, especially since it has been implicated as a significant contributor to the alarming increase in the incidence of several metabolic disorders, including obesity, non-alcoholic fatty liver disease, and cardiovascular diseases.[4–6]

One of the earliest reports of adverse metabolic effects from fructose consumption was the discovery of hereditary fructose intolerance in the 1950s by Chambers and Pratt.[7] Since this early finding, a wide variety of fructose-induced adverse metabolic effects have been reported in both animals and humans. For instance, fructose has been shown to induce lactic acidosis, high blood pressure, *de novo* lipogenesis, oxidative stress, and impairment of insulin sensitivity.[8–13] Recent studies have emphasized the importance of the rapid metabolism of fructose in the liver by ketohexokinase (KHK) as the key mechanism driving fructose-induced adverse metabolic effects.[14–18]

After dietary fructose is absorbed into the portal vein from the intestinal lumen, it is metabolized by the liver through a specific pathway consisting of three enzymes–KHK, aldolase B, and triokinase. Fructose is initially phosphorylated by KHK into fructose-1-phosphate which is further converted to lactate, glucose, and fatty acids.[19] Due to the lack of a negative feedback mechanism, fructose is rapidly metabolized. This causes an acute depletion of hepatic ATP levels which leads to increased production of uric acid, as well as, other downstream adverse effects.[19–25] Studies have shown that blocking the activity of KHK ameliorates the harmful effects of fructose. In a cell culture study using KHK knockdown by shRNA, kidney proximal tubular cells were protected against fructose-induced inflammation and oxidative stress.[15] In addition, studies examining the effects of a high fructose diet showed that KHK-knockout mice were protected against fructose-induced fatty liver, weight gain, inflammation, and insulin resistance.[17, 18]

**Fig 1:**
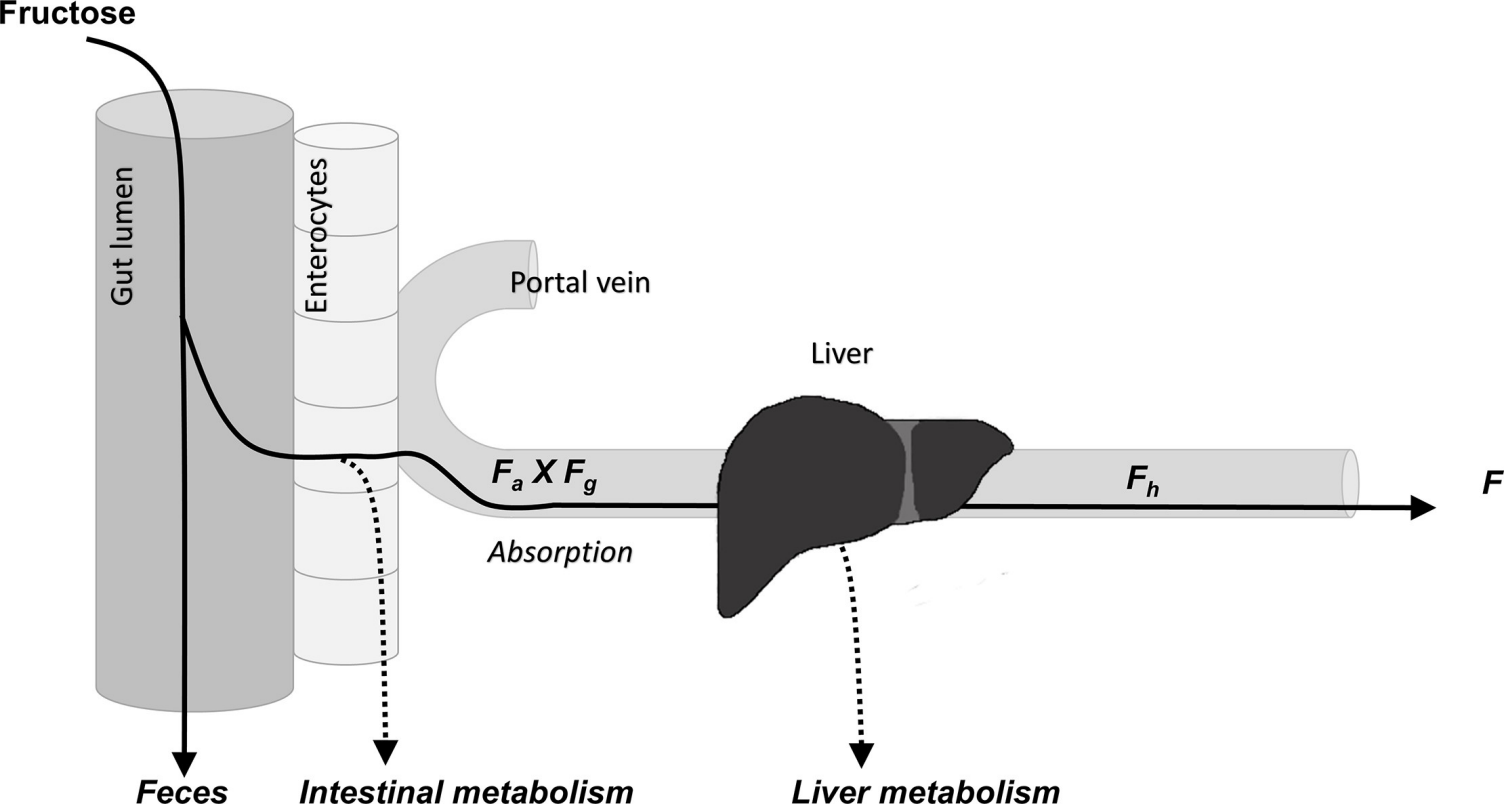
Fructose intestinal, hepatic, and oral bioavailability. F_a_ x F_g_ = intestinal availability or fructose absorption. F_h_ = hepatic availability. F = oral bioavailability.

Because the liver plays an important role in the unregulated and rapid metabolism of fructose, assessing the intestinal and hepatic availability of fructose is critical to better understanding the metabolic response to fructose, and thus, the susceptibility for developing fructose-induced adverse effects. The oral bioavailability of fructose represents the fraction of the unmetabolized dose that reaches the systemic circulation after it has been absorbed through the gut and undergone first pass metabolism by the intestine and the liver (Fig 1). Thus, intestinal availability represents fructose absorption, the fraction of the fructose dose that was absorbed through the gut and reaches unmetabolized into the portal vein. Hepatic availability is the fraction of the absorbed fructose dose that is not metabolized by the liver. Therefore, for this study, dual-catheterized rats were used, facilitating simultaneous and serial sampling of the portal and systemic circulations, thus allowing more accurate assessment of fructose absorption and hepatic metabolism.[26]

One factor that can potentially influence an individual’s response to fructose is the composition of fructose-containing sweeteners. As a simple sugar, fructose can be consumed from fruits and vegetables. However, the major source of dietary fructose comes from added sugars, especially as sucrose or high-fructose corn syrup. Sucrose is a disaccharide of glucose and fructose and is broken down in the intestine by sucrase. High-fructose corn syrup consists of monosaccharides, typically, consisting of 55% fructose and the rest being primarily glucose.[1] Therefore, it has been proposed that the free fructose from high-fructose corn syrup is more readily absorbed through the gut, and thus, have different health effects when compared to sucrose.[1, 27] Other studies have suggested that glucose can enhance the absorption of fructose, which would increase the amount of fructose available to be metabolized.[28–34] Thus, we hypothesize that fructose, when co-ingested with glucose, as with sucrose or high fructose corn syrup, results in greater absorption and higher amounts of fructose being metabolized in the liver. Chronically, this could possibly lead to greater adverse effects related to fructose. Although previous studies have suggested that the composition of different fructose-containing sweeteners can affect fructose bioavailability, few studies have directly compared these sweeteners and their impact on fructose levels.[35, 36] Thus, the objective of this study was to characterize the impact of different fructose-containing sweeteners on the absorption of fructose and its hepatic metabolism.

## Materials and methods

### Dual-catheterized rats

Male Wistar rats (~250 g) were purchased from Harlan Laboratories (Madison, WI) with catheters surgically implanted in both the portal and femoral veins. The patency of the catheters was maintained with regular replacement of the lock solution (Taurolidine-citrate catheter solution, Access Technologies, Skokie, IL). Rats were allowed 1 week to acclimate to the new environment and were fed a standard chow diet. All procedures performed on the animals were approved by the Institutional Animal Care and Use Committee at the University of Colorado Denver, Anschutz Medical Campus. Animals were euthanized by using isoflurane followed by bilateral thoracotomy. These methods are consistent with the recommendations of the Panel on Euthanasia of the American Veterinary Medical Association. The approved animal protocol number is 86215(08)1D.

### Sugar treatments and blood collections

The rats were gavaged with 1 mL of a starch liquid diet (Harlan TD.120513) then placed in metabolic cages for overnight fasting (~16 hr). Rats (n = 8–10) were then randomized to a treatment group and gavaged with 1 mL of a carbohydrate solution of fructose, 45% glucose/55% fructose (45/55 G/F) which is representative of high fructose corn syrup, sucrose, glucose, or tap water. The total carbohydrate dose (1 mg/g) in each treatment was 250 mg (Table 1). The fructose dose was 250 mg for the fructose solution, 137.5 mg for the 45/55 G/F solution, and 125 mg for the sucrose solution. These carbohydrate doses emulate doses used in other human studies (1–2 g sugar/kg body weight).[30–34, 37] Blood samples from unanesthetized rats were drawn simultaneously from the portal and femoral veins at the following time points: 0 (before gavage), 15, 30, 60, 120, 240, and 360 min. Serum samples were then separated by centrifugation in serum separator tubes and stored at -80°C.

**Table 1:** Total carbohydrate and fructose dose and estimated fructose amounts after absorption and metabolism.

Treatment	Gavage Volume	Total Carbohydrate Dose	Fructose Dose	Post Intestinal Fructose	Post Hepatic Fructose	Systemic Fructose
	(mL)	(mg)	(mg)	(mg)	(mg)	(mg)
Fructose	1	250	250	154.3	50.9	47
45/55 G/F	1	250	137.5	72.5	32.7	32
Sucrose	1	250	125	70.6	31.9	30
Glucose	1	250	-	-	-	-
Water	1	-	-	-	-	-

45/55 G/F = 45% glucose/55% fructose.

### Measurements

Fructose levels were measured using the EnzyChrom™ Fructose Assay Kit (BioAssay Systems, Hayward, CA). The samples were analyzed in duplicate with and without enzyme to account for background absorbance. A Synergy 2 multi-mode microplate reader was used to measure fructose levels at A_565nm_ (BioTek Instruments, Inc., Winooski, VT).

### Whole blood to plasma fructose concentration ratio

Fresh blood samples from three untreated rats were used to determine the whole blood to plasma concentration ratio of fructose. Blood samples were collected in blood collection tubes containing lithium heparin. The samples were spiked with a final concentration of 500 μM fructose then incubated at 37°C for 15 min.[38] Plasma and red blood cells were separated by centrifugation at 3,000 x g for 10 min at room temperature. Plasma samples were collected and measured for fructose levels. Plasma and serum have been shown to have similar fructose concentrations.[39] To measure fructose levels in red blood cells, 100 uL of red blood cells were washed twice using two volumes of cold saline and collected after centrifugation at 3,000 x g for 5 min at 4°C. To lyse the red blood cells, 300 μL cold water was added and the samples were vigorously vortexed and then placed at -80°C for 15 min. 300 μL of a solution consisting of 62.5% ethanol and 37.5% chloroform was added to the erythrocyte lysates to remove hemoglobin. The samples were vigorously vortexed for 15 min and centrifuged at 2,500 rpm for 10 min at 4°C. The water-ethanol layers were collected and measured for fructose levels.[26, 40, 41] To determine the % hematocrit (Hct), the packed cell volume and plasma ratio was measured from whole blood that was collected in a heparinized capillary tube and then centrifuged for 2 min.

The whole blood to plasma concentration ratio (K_b/p_) for fructose was calculated using the following equation:

$$K_{b/p}=K_{e/p}\times Hct+(1-Hct)$$ [Eq 1]

K_e/p_ is the red blood cell to plasma partition coefficient. K_e/p_ is the ratio of the concentration of the compound in red blood cells (C_RBC_) over plasma (C_PL_).[38, 42]

$$K_{e/p}=\frac{C_{RBC}}{C_{PL}}$$ [Eq 2]

### Pharmacokinetic analysis

WinNonlin 6.3 (Pharsight Corporation, Mountain View, CA) was used to calculate the following pharmacokinetic (PK) parameters: area under the curve (AUC) of serum concentration versus time, maximum observed concentration (C_max_), and elimination half-life (T_1/2_). To adjust for the endogenous fructose levels of each animal, the C_max_ was adjusted (AdjC_max_) by subtracting the fructose concentration at baseline (time = 0) from C_max_. Fructose AUC and AdjC_max_ were normalized to the fructose dose of the sugar solutions and the body weight of the animal. Thus, AUC/D and AdjC_max_/D represent the data at a dose of 1 mg/g. Noncompartmental analyses were conducted using the linear/log trapezoidal calculation method.

Oral bioavailability of fructose (*F*) was calculated as follows (illustrated in Fig 1):

$$F=F_{a}\times F_{g}\times F_{h}$$ [Eq 3]

*F*_*a*_ was the fraction of the dose that was absorbed from the gastrointestinal (GI) tract into the enterocytes. *F*_*g*_ was the fraction of the dose that remained after intestinal metabolism and *F*_*h*_ represented the fraction of the dose that remained after being metabolized in the liver and reaches the systemic circulation.[26]

By evaluating the difference between portal and systemic blood concentrations of fructose after oral dosing, fructose absorption or intestinal availability (*F*_*a*_×*F*_*g*_) was calculated by using Eq 4.

$$F_{a}\times F_{g}=Q_{pv}\times K_{b/p}\times \left({AUC}_{pv}-{AUC}_{sys}\right)/D$$ [Eq 4]

Q_pv_ is the portal blood flow, K_b/p_ is the whole blood to plasma concentration ratio, AUC_pv_ is the AUC in the portal vein, AUC_sys_ is the AUC in the systemic circulation, and D is the fructose dose, adjusted to the body weight of each animal. Thus, *F*_*a*_×*F*_*g*_ is normalized at a dose of 1 mg/g.[26, 43] The value of Q_pv_ in rats was estimated to be 32.9 ml/min/kg.[26]

Hepatic availability (*F*_*h*_) was calculated using Eq 5.[44]

$$F_{h}={AUC}_{sys}/{AUC}_{pv}$$ [Eq 5]

The hepatic extraction ratio (E_h_) represents the fraction of the dose metabolized by the liver and is calculated using Eq 6.

$$E_{h}=1-F_{h}$$ [Eq 6]

### Data analysis

Two-tailed unpaired t-tests were conducted to compare the fructose-containing sweeteners and to compare fructose to the glucose and water controls. P-values <0.05 were considered significant. If the p-value of the equality of variances test was smaller than 0.10, the Satterthwaite test was employed. All analyses were performed using SAS 9.4 (SAS Institute Inc., Cary, NC, USA).

## Results

### The absorption of fructose is rapid and concludes quickly

**Fig 2:**
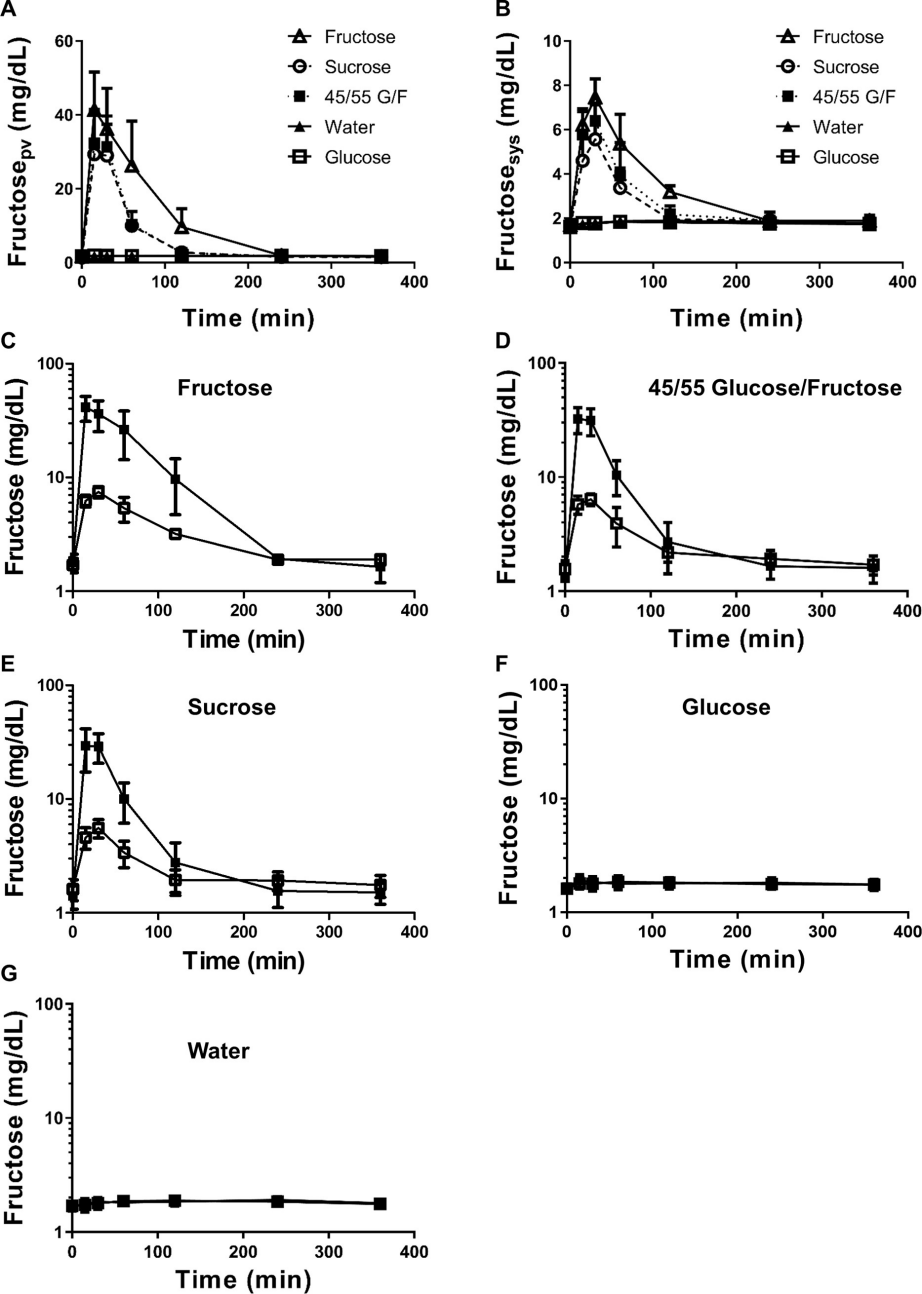
Fructose serum concentration versus time profiles after oral administration of sugar-sweetened solutions. A) Fructose concentrations in the portal vein over 6-hr. B) Fructose concentrations in the systemic circulation (femoral vein) over 6-hr. Wistar rats were gavaged with 1 mL of the following solutions: (C) Fructose, (D) 45/55 Glucose/Fructose, (E) Sucrose, (F) Glucose, and (G) Water. Portal serum concentrations are shown as closed symbols. Systemic serum concentrations are shown as open symbols. Data represents the mean ± standard deviation.

Fig 2 and S1 Table shows the portal and systemic serum concentrations of fructose after the oral administration of a 1 g/kg solution of fructose, 45/55 G/F, sucrose, glucose, or water. From the semi-log plots of the portal and systemic serum concentration-time profiles (Fig 2C–2E), the fructose concentrations in the terminal phase overlapped during the six-hour study. The overlapping patterns indicate that the absorption rate constant of fructose is higher than its elimination rate constant (k_a_ > k_e_).[26] The analysis of both portal and systemic serum concentrations in the catheterized rats eliminated the need for a bolus intravenous dose of fructose to account for possible flip-flop kinetics. Thus, the absorption of fructose from the intestine was rapid and concluded quickly, resulting in minimal impact of absorption during the terminal phase.

### Glucose does not enhance fructose absorption

**Fig 3:**
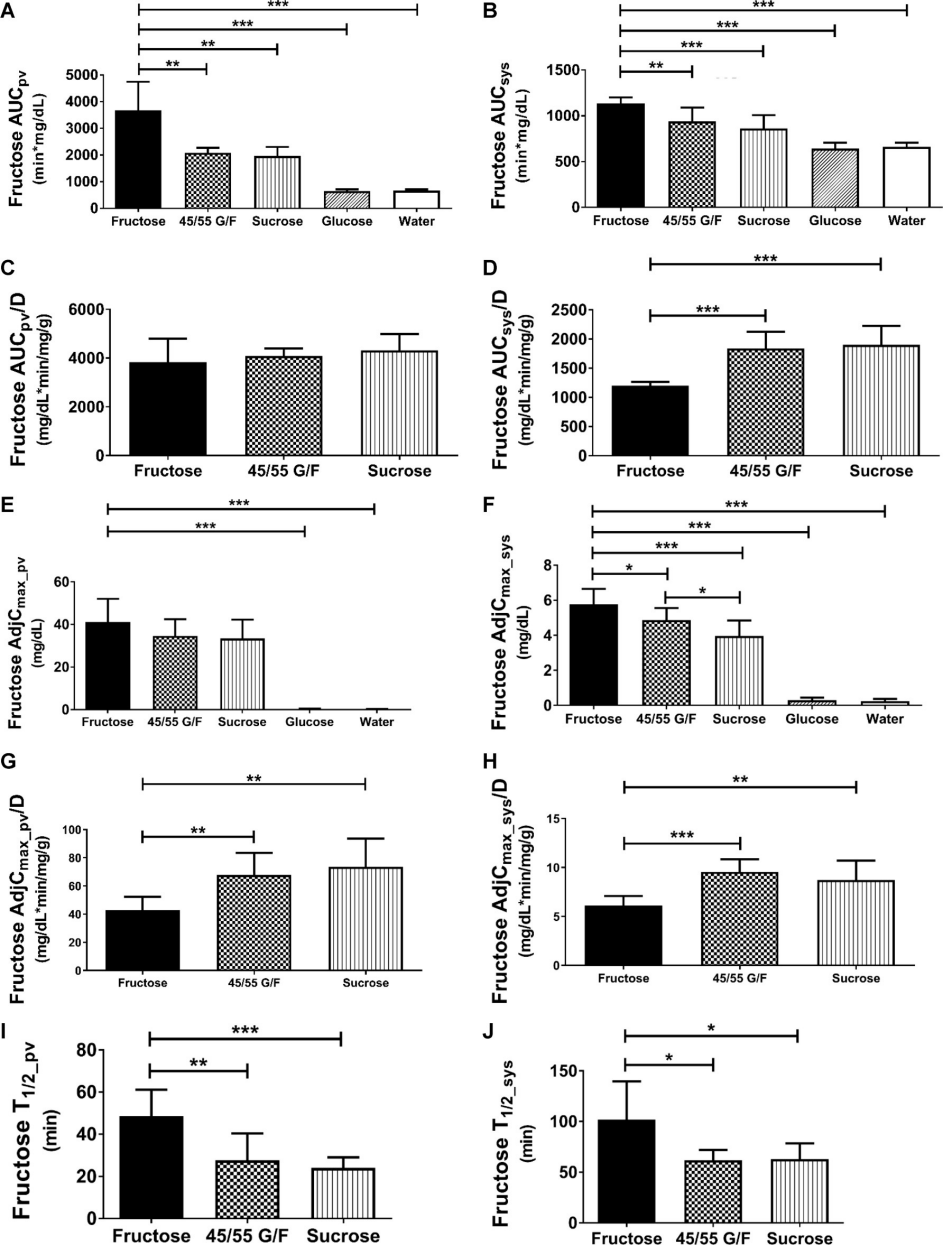
Effects of sugar-sweetened solutions after oral administration on fructose pharmacokinetic parameters. (A) Portal fructose area under the curve (AUC_pv_) (B), systemic fructose area under the curve (AUC_sys_), (C) normalized portal fructose area under the curve (AUC_pv_/D), (D) normalized systemic fructose area under the curve (AUC_sys_/D), (E) adjusted portal fructose maximal concentration (AdjC_max_pv_), (F) adjusted systemic fructose maximal concentration (AdjC_max_sys_), (G) normalized adjusted portal fructose maximal concentration (AdjC_max_pv_/D), (H) normalized adjusted systemic fructose maximal concentration (AdjC_max_sys_/D), (I) portal fructose half-life (T_1/2_pv_), and, (J) systemic fructose half-life (T_1/2_sys_). 45/55 G/F = 45% glucose/55% fructose. P-value: * < 0.05, ** < 0.01, and *** < 0.001.

**Fig 4:**
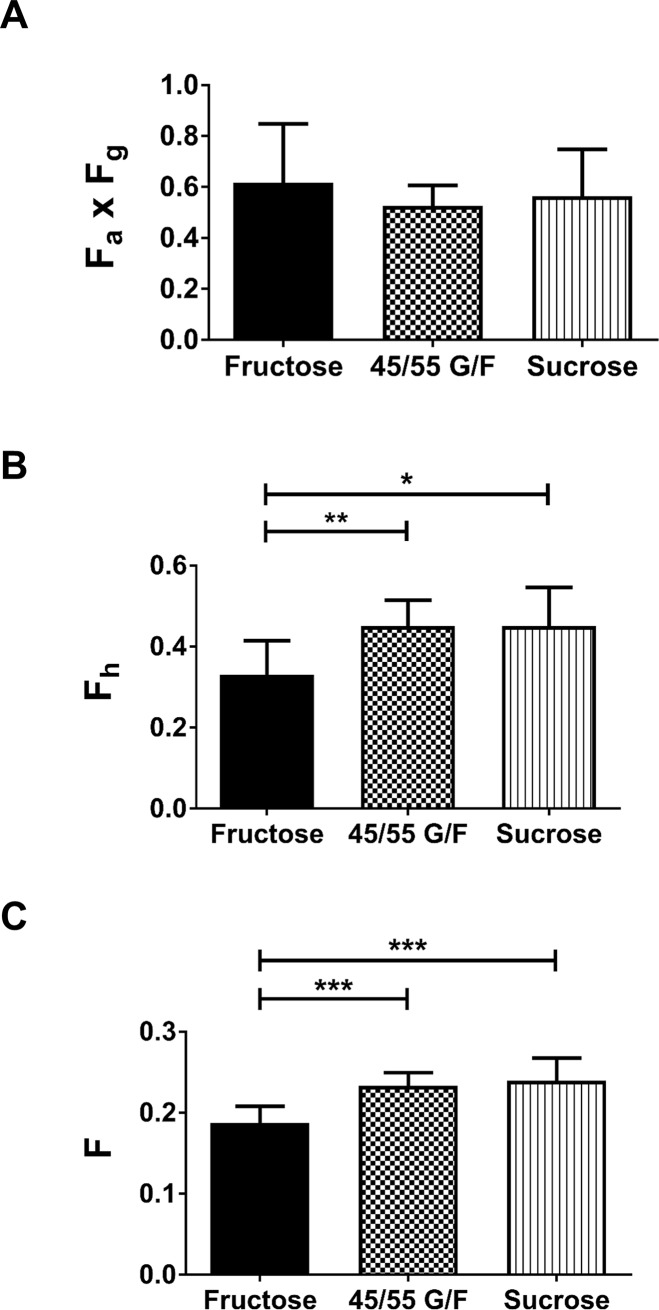
**Effects of sugar-sweetened solutions** after oral administration on (A) intestinal fructose bioavailability (F_a_ x F_g_), (B) hepatic fructose bioavailability (F_h_), and (C) systemic fructose bioavailability (F). 45/55 G/F = 45% glucose/55% fructose. P-value: * < 0.05, ** < 0.01, and *** < 0.001.

Figs 3 and 4 and Table 2 and S2–S4 Tables compares the PK parameters of fructose within the portal and systemic circulations of the rats. After oral administration of the 1 g/kg sugar solutions, the portal AUC represents the body’s total fructose exposure after it is absorbed from the intestine. Fructose-gavaged animals had over 40% higher portal AUC than 45/55 G/F or sucrose (Fig 3A), which can be attributed to the higher fructose dose. Once the AUCs were normalized to the fructose doses, AUC_pv_/D were similar for all three fructose-containing sweeteners (Fig 3C). This suggests that fructose was absorbed at a similar rate regardless of the fructose dose or carbohydrate formulation. This is supported by our calculation of intestinal availability which reflects the unmetabolized fraction of the fructose dose that was absorbed from the intestine and entered the portal vein. Based on Eq. 4 which takes into account fructose’s whole blood to plasma concentration ratio (K_b/p_ = 0.711 ± 0.026; S5 Table) and its systemic circulation, *F*_*a*_×*F*_*g*_ of fructose after oral administration of fructose, 45/55 G/F, or sucrose were 0.62, 0.53 and 0.57, respectively. This indicates that about 50–60% of the fructose was absorbed from the GI tract (Table 2, Fig 4A). Thus, 40–50% of the fructose doses were lost due to intestinal metabolism, bacterial metabolism in the GI tract, or through fecal matter. Measuring fructose amounts in the feces would have allowed us to determine the fructose dose metabolized by the intestine. However, in order to distinguish between enterocyte fructose metabolism and microbiome fructose metabolism, a germ-free environment is necessary. Overall, our data shows that the co-ingestion of glucose does not enhance fructose absorption into the portal vein.

**Table 2:** Fructose pharmacokinetic parameters after oral administration of sugar-sweetened solutions.

Parameter	Fructose	45%/55% Glucose/Fructose	Sucrose	Glucose	Water
	(n = 8)	(n = 10)	(n = 9)	(n = 8)	(n = 8)
AUC_pv_ (min*mg/dL)	3672.2 ± 1074.3	2087.2 ± 185.2^A^**	1961.7 ± 344.9^B^**	647.2 ± 66.3^C^***	591.1 ± 109.5^D^***
AUC_pv_/D (min*mg/dL/mg/g)	3837.7 ± 964.8	4091.8 ± 311.9	4313.3 ± 677.7	-	-
AUC_sys_ (min*mg/dL)	1138.7 ± 63.4	939.1 ± 150.9^A^**	861.7 ± 146.3^B^***	642.2 ± 64.7^C^***	620.9 ± 70.3^D^***
AUC_sys_/D (min*mg/dL/mg/g)	1200.1 ± 66.4	1841.3 ± 284.6^A^***	1900.2 ± 325.4^B^***	-	-
AdjC_max_pv_ (mg/dL)	41.14 ± 10.96	34.54 ± 7.92	33.39 ± 8.89	0.30 ± 0.18^C^***	0.34 ± 0.39^D^***
AdjC_max_pv_/D (min*mg/dL/mg/g)	42.96 ± 9.39	67.84 ± 15.74^A^**	73.66 ± 19.98^B^**	-	-
AdjC_max_sys_ (mg/dL)	5.77 ± 0.88	4.87 ± 0.68^A^*	3.96 ± 0.89^B^***^, E^*	0.29 ± 0.16^C^***	0.32 ± 0.25^D^***
AdjCmax_sys/D (min*mg/dL/mg/g)	6.09 ± 1.00	9.55 ± 1.29^A^***	8.73 ± 1.98^B^**	-	-
T_1/2_pv_ (min)	48.5 ± 12.6	27.5 ± 12.8^A^**	24.0 ± 4.95^B^***	-	-
T_1/2_sys_ (min)	101.8 ± 37.8	61.5 ± 10.4_A_*	62.8 ± 15.6^B^*	-	-
F_a_ x F_g_	0.617 ± 0.231	0.527 ± 0.080	0.565 ± 0.183	-	-
F_h_	0.330 ± 0.085	0.451 ± 0.064^A^**	0.451 ± 0.096^B^*	-	-
F	0.188 ± 0.021	0.233 ± 0.016^A^***	0.240 ± 0.028^B^***	-	-
E_h_	0.670 ± 0.085	0.549 ± 0.064^A^**	0.550 ± 0.096^B^*	-	-

AdjC_max_ = maximum observed concentration—concentration at time = 0. AUC = area under the curve. AUC/D = area under the curve normalized by fructose dose. E_h_ = hepatic extraction ratio. F = systemic bioavailability. F_a_ X F_g_ = intestinal availability. F_h_ = hepatic availability. PV: portal vein. SYS: femoral vein. A: fructose vs 45%/55% glucose/fructose. B: fructose vs sucrose. C: fructose vs glucose. D: fructose vs water. E: 45%/55% glucose/fructose vs sucrose. P-value:

* < 0.05

** < 0.01, and

*** < 0.001.

### Glucose reduces hepatic metabolism of fructose

The hepatic availability of fructose, which represents the fraction of the absorbed dose that remained after being metabolized in the liver and reaching the systemic circulation, was estimated based on the portal and systemic AUC (Eq. 5). Thus, *F*_*h*_ for the fructose solution was 0.33, 0.45 for 45/55 G/F, and 0.45 for sucrose (Fig 4B, Table 2). This indicates that fructose was metabolized more rapidly when it was administered as a fructose-only solution than as a 45/55 G/F or sucrose solution. From the fructose-only solution, about 67% of the fructose that was absorbed and reached the portal vein was broken down in the liver, compared to the hepatic extraction ratio of fructose from 45/55 G/F and sucrose solutions which was 55% (Table 2). Based on the values of *E*_*h*_, the metabolism of fructose by the liver is considered to be intermediate (*E*_*h*_ = 0.3–0.7).[45] Although the half-life of fructose in the portal and systemic circulations was about two times longer from the fructose-only solution compared to the 45/55 G/F and sucrose solutions, this is most likely influenced by the higher fructose dose and greater amount of fructose absorbed (Fig 3I and 3J).

The increased metabolism of fructose in the body from the fructose solution can also be seen by the significantly lower systemic AUC and adjusted C_max_ after normalizing for the fructose doses (Fig 3D and 3H). Both 45/55 G/F and sucrose had about a 50% higher fructose AUC_sys_/D and AdjC_max_sys_/D compared to the fructose solution. As a result of the higher fructose metabolism, the systemic oral bioavailability of fructose (Fig 4C, Table 2) from the fructose solution was 0.19 which was significantly lower than from 45/55 G/F (*F* = 0.23) and from sucrose (*F* = 0.24). Therefore, only about 47 mg of the original 250 mg fructose dose from the fructose solution reached the systemic circulation (Table 1). For 45/55 G/F, about 32 mg of the 137.5 mg fructose dose reached the systemic circulation. For sucrose, about 30 mg of the 125 mg fructose dose reached the systemic circulation. Overall the data shows that the co-ingestion of glucose decreases hepatic metabolism of fructose and potentially its metabolism in other tissues.

### Fructose absorption and metabolism are similar between 45/55 G/F and sucrose

The 45/55 G/F had a 10% higher fructose dose compared to the sucrose solution. The small differences in the fructose PK parameters between these two sweeteners reflected the small difference in the fructose dose. Both 45/55 and sucrose exhibited similar intestinal absorption and hepatic metabolism of fructose, resulting in similar oral systemic bioavailability (Table 2, Figs 3 and 4). For animals gavaged with 45/55 G/F, there was about a 6% greater difference in the portal and systemic AUC when compared to sucrose-gavaged animals (Fig 3A and 3B). 45/55 G/F had about 3% higher AdjC_max_pv_ and about 18% higher AdjC_max_sys_ than sucrose (Fig 3E and 3F). However, once normalized for the fructose doses, the differences between the sweeteners were minimal. The data suggests that the breakdown of sucrose by sucrase in the GI tract is rapid and does not hinder the absorption of fructose when compared to free molecules of fructose found in high fructose corn syrup. However, polymorphisms of sucrase that impact its activity could affect the absorption of fructose from sucrose.[46]

## Discussion

The liver plays an essential role in driving the adverse effects of fructose by rapidly metabolizing it.[14–18] To gain a better understanding of the liver’s exposure to dietary fructose and its potential toxicities, the intestinal absorption and hepatic metabolism of fructose needs to be assessed. Thus, to advance our understanding of whether the formulation of fructose-containing sweeteners can impact fructose absorption and metabolism, our study compared the effects of fructose, 45/55 G/F, and sucrose on the intestinal, hepatic, and oral bioavailability of fructose. At 1 g/kg carbohydrate dose, we found that the formulation of fructose-containing sweeteners did not have an impact on fructose absorption since all three sweeteners had similar percentages of fructose absorption, which was approximately 50–60% of the fructose dose. However, the formulation of the sweeteners did impact fructose metabolism. The co-ingestion of glucose decreased the metabolism of fructose, which increased the hepatic availability and oral bioavailability of fructose for both 45/55 G/F and sucrose versus fructose. The intestinal, hepatic, and oral bioavailability of fructose was similar between 45% glucose/55% fructose and sucrose. While these studies demonstrated the acute impact of sweetener formulations, future studies are needed to evaluate the effects of chronic, diet-induced regulation of fructose absorption and metabolism. In addition, further studies are needed to evaluate whether lower carbohydrate doses impact fructose bioavailability differently.

Studies that have directly measured fructose levels have shown that the intestine plays an important role in fructose metabolism by converting it into glucose, lactate, and glycerate, thus, highly impacting the amount of fructose available for metabolism by the liver and other organs.[47–49] Factors, such as diets, have been shown to impact fructose absorption.[49–52] For instance, in a study comparing the effects of high carbohydrate diets on fructose and glucose absorption, it was shown that the consumption of a high fructose or high sucrose diets, but not a high glucose diet, stimulated intestinal fructose absorption in rats.[52] In addition, after being exposed to a high glucose and fructose diet for 3 days, the intestinal absorption of fructose was increased indicated by higher levels of fructose in the systemic circulation.[49] It was also shown that at low fructose doses the intestine is responsible for metabolizing about 90% of the fructose dose. In addition, mice in a fed state had greater intestinal metabolism of fructose compared to the fasted state. Overall, these studies show that the intestinal absorption of fructose can greatly vary. This emphasizes the need to measure intestinal availability, the amount of fructose that is available for metabolism by the liver, which is critical to our understanding of what factors can influence fructose-induced adverse effects in the body.

One of the most surprising findings from our study is the inability of glucose to enhance the absorption of fructose. Previous studies have suggested that the co-ingestion of glucose could enhance the absorption of fructose, and thus, reduce gastrointestinal issues, such as abdominal pain, diarrhea, nausea, and vomiting, from fructose malabsorption.[30–34, 53] Because of the slower intestinal transport rate of fructose compared to glucose, the prevalence of fructose malabsorption is high.[20, 54–56] Fructose malabsorption is typically diagnosed using the hydrogen breath test, a noninvasive measurement based on the concept that gas produced by colonic bacterial fermentation of unabsorbed carbohydrates diffuses into the blood and is excreted by breath, where it can be quantified easily by chromatography.[57, 58] After the ingestion of a fructose load, individuals with breath hydrogen levels ≥ 20 ppm are considered positive for fructose malabsorption. About 50–70% of infants and children that were given a fructose dose of 1–2 g/kg body weight, up to 50 g, were shown to be malabsorbers.[30, 31, 59] Meanwhile, about 35–80% of healthy adults that ingested 50 g of fructose were malabsorbers.[32–34, 37] Interestingly, if fructose was consumed with glucose or given as a sucrose load, the breath hydrogen levels were greatly improved. This has led to the assumption that glucose enhances the absorption of fructose. However, recent studies have shown that the hydrogen breath test is unreliable. The production of hydrogen gas can be greatly affected by differences in oxidation rate of sugars, absorption rate of sugars, rate of colonic fermentation, or colonic activity and bacterial populations.[60–64] The test may also result in false negative measurements in hydrogen nonexcretors who produce methane gas instead of hydrogen.[65] In addition, the results of a hydrogen breath test can be affected depending on the fructose load used for the test.[37]

The impacts fructose has on various diseases and health disorders have been poorly understood due to conflicting or inconclusive data. For instance, some studies have associated fructose malabsorption with bowel disorders [66, 67]. Nevertheless, some irritable bowel syndrome patients developed intestinal issues although they had normal breath results.[68] The breath test also failed to distinguish patients benefiting from fructose-reduced diets.[69] In addition, studies have shown that the simultaneous ingestion of glucose with fructose may enhance fructose transport and prevent malabsorption gastrointestinal effects.[70, 71] Yet, other studies have reported that inconclusive results did not support the effects of glucose on fructose transport, and thus, should not be suggested as an effective strategy to reduce fructose-related gastrointestinal issues.[72] A potential issue is that these studies based their analyses on the erratic outcomes of the hydrogen breath test when determining fructose intestinal absorption, and thus, there is a need for a more accurate methodology.

In our study, the use of dual-catheterized rats allowed for the simultaneous and unrestrained serial sampling of systemic and portal blood after an oral carbohydrate dose.[26, 73] There are several benefits when using this technique. First, the method eliminates the use of anesthesia. Studies have shown that anesthesia can significantly affect the body’s ability to absorb and metabolize.[74, 75] For instance, pentobarbital was shown to inhibit the hepatic metabolism of oxacillin, where conscious rats eliminated about 90% of oxacillin while anesthetized rats metabolized only 60%.[76] Second, by sampling blood from the portal and femoral vein, we were able to circumvent factors such as blood flow rate.[43] However, it is also important to note that while hepatic vein catheterization would be a more direct method to measure liver metabolism, this technique is technically challenging. Nonetheless, by directly sampling from the portal vein and from the femoral vein, we were able to measure levels of unmetabolized fructose, and more accurately determine fructose absorption through the GI tract, metabolism in the liver, and systemic oral bioavailability. Our results showed that fructose levels in the portal and systemic circulation were similar to previously published studies that used catheterization in animals and humans.[77, 78] In addition, our results demonstrated comparable metabolism of fructose by the liver in a catheterized rat.[79]

In conclusion, our study showed that there were no differences in fructose absorption between fructose, 45/55 G/F, and sucrose. Although the fractions absorbed were similar, the total amount of fructose absorbed was higher from the fructose-only treatment. Further studies are needed to determine if the greater exposure to fructose in the liver leads to greater adverse effects. 45/55 G/F and sucrose had similar percentages of fructose hepatic metabolism. Nevertheless, when compared to fructose-gavaged animals, the hepatic metabolism of fructose from 45/55 and sucrose was over 10% lower. Our results support a previous finding that showed that the co-ingestion of glucose decreased fructose oxidation.[71] Importantly, the higher fructose metabolism from the fructose-only treatment implies that fructose can also be metabolized at a faster rate in the intestine and potentially in other organs, similar to the liver. Overall, we showed that glucose does not enhance fructose absorption, contrary to the assumptions of previously published studies, but it does have an effect on fructose metabolism. By utilizing dual-catheterized rats, further studies can be conducted to elucidate the impact of various factors, such as the interplay between diet and the microbiome, on fructose absorption and metabolism and, more importantly, whether these factors impact the susceptibility to develop fructose-induced adverse effects.

## Supporting information

S1 Table Data for fructose concentration versus time in femoral and portal veins. (PDF)

S2 Table Data for fructose AUC and T_max_ in femoral and portal veins. AUC = area under the curve. PV: portal vein. SYS: femoral vein. T_max_ = time at maximum observed concentration.
(PDF)

S3 Table Data for fructose AdjC_max_ in femoral and portal veins. AdjC_max_ = maximum observed concentration—concentration at time = 0. C_max_ = maximum observed concentration.
(PDF)

S4 Table Data for fructose AdjCmax/D, AUC/D, and bioavailabilty. AdjC_max_/D = adjusted maximum observed concentration normalized by fructose dose. AUC/D = area under the curve normalized by fructose dose. BW = body weight at sacrifice. D = fructose amount/body weight. E_h_ = hepatic extraction ratio. F = systemic bioavailability. F_a_ x F_g_ = intestinal availability. F_h_ = hepatic availability. K_b/p_ = whole blood to plasma concentration ratio. PV: portal vein. Q_pv_ = portal blood flow. SYS: femoral vein.
(PDF)

S5 Table Data for whole blood to plasma ratio of fructose. CRBC = concentration in red blood cells. CPL = concentration in plasma. K_b/p_ = whole blood to plasma concentration ratio.
(PDF)
