# Supplementary material for: Effects of fructose-containing sweeteners on fructose intestinal, hepatic, and oral bioavailability in dual-catheterized rats
Source: PLoS One. 2018 Nov 8;13(11):e0207024. doi: 10.1371/journal.pone.0207024 (PMC6224110; doi:10.1371/journal.pone.0207024)
Supplement: S1 Table — (PDF) [file pone.0207024.s001.pdf]

S1 Table. Data for Fructose Concentration versus Time in Femoral and Portal Veins.

Rat ID	Gavage Treatment	Vein	Time (min)	Fructose (mg/dL)
Rat #102	Water	Femoral	0	1.47
Rat #102	Water	Femoral	15	1.53
Rat #102	Water	Femoral	30	1.63
Rat #102	Water	Femoral	60	1.81
Rat #102	Water	Femoral	120	1.77
Rat #102	Water	Femoral	240	1.87
Rat #102	Water	Femoral	360	1.66
Rat #105	Water	Femoral	0	1.69
Rat #105	Water	Femoral	15	1.82
Rat #105	Water	Femoral	30	1.83
Rat #105	Water	Femoral	60	1.98
Rat #105	Water	Femoral	120	1.82
Rat #105	Water	Femoral	240	1.81
Rat #105	Water	Femoral	360	1.68
Rat #108	Water	Femoral	0	1.61
Rat #108	Water	Femoral	15	1.42
Rat #108	Water	Femoral	30	1.80
Rat #108	Water	Femoral	60	2.06
Rat #108	Water	Femoral	120	2.02
Rat #108	Water	Femoral	240	2.01
Rat #108	Water	Femoral	360	2.03
Rat #112	Water	Femoral	0	1.70
Rat #112	Water	Femoral	15	1.87
Rat #112	Water	Femoral	30	1.72
Rat #112	Water	Femoral	60	1.81
Rat #112	Water	Femoral	120	1.90
Rat #112	Water	Femoral	240	1.93
Rat #112	Water	Femoral	360	1.85
Rat #114	Water	Femoral	0	1.68
Rat #114	Water	Femoral	15	1.67
Rat #114	Water	Femoral	30	1.63
Rat #114	Water	Femoral	60	1.79
Rat #114	Water	Femoral	120	1.70
Rat #114	Water	Femoral	240	1.72
Rat #114	Water	Femoral	360	1.76
Rat #117	Water	Femoral	0	1.98
Rat #117	Water	Femoral	15	2.12
Rat #117	Water	Femoral	30	2.21
Rat #117	Water	Femoral	60	1.98
Rat #117	Water	Femoral	120	2.23
Rat #117	Water	Femoral	240	1.85
Rat #117	Water	Femoral	360	1.80
Rat #120	Water	Femoral	0	1.90
Rat #120	Water	Femoral	15	1.91
Rat #120	Water	Femoral	30	1.96
Rat #120	Water	Femoral	60	1.89
Rat #120	Water	Femoral	120	1.97
Rat #120	Water	Femoral	240	1.98
Rat #120	Water	Femoral	360	1.80
Rat #123	Water	Femoral	0	1.57
Rat #123	Water	Femoral	15	1.54
Rat #123	Water	Femoral	30	1.59
Rat #123	Water	Femoral	60	1.68
Rat #123	Water	Femoral	120	1.66
Rat #123	Water	Femoral	240	1.66
Rat #123	Water	Femoral	360	1.59
Rat #204	Sucrose	Femoral	0	1.32
Rat #204	Sucrose	Femoral	15	4.44
Rat #204	Sucrose	Femoral	30	4.41
Rat #204	Sucrose	Femoral	60	2.80
Rat #204	Sucrose	Femoral	120	1.46
Rat #204	Sucrose	Femoral	240	1.42
Rat #204	Sucrose	Femoral	360	1.39
Rat #205	Sucrose	Femoral	0	1.86

S1 Table. Data for Fructose Concentration versus Time in Femoral and Portal Veins.

Rat ID	Gavage Treatment	Vein	Time (min)	Fructose (mg/dL)
Rat #205	Sucrose	Femoral	15	5.10
Rat #205	Sucrose	Femoral	30	6.52
Rat #205	Sucrose	Femoral	60	2.36
Rat #205	Sucrose	Femoral	120	1.53
Rat #205	Sucrose	Femoral	240	1.59
Rat #205	Sucrose	Femoral	360	1.45
Rat #211	Sucrose	Femoral	0	1.36
Rat #211	Sucrose	Femoral	15	3.82
Rat #211	Sucrose	Femoral	30	4.36
Rat #211	Sucrose	Femoral	60	2.10
Rat #211	Sucrose	Femoral	120	1.55
Rat #211	Sucrose	Femoral	240	1.49
Rat #211	Sucrose	Femoral	360	1.22
Rat #221	Sucrose	Femoral	0	1.84
Rat #221	Sucrose	Femoral	15	5.53
Rat #221	Sucrose	Femoral	30	6.89
Rat #221	Sucrose	Femoral	60	3.77
Rat #221	Sucrose	Femoral	120	2.45
Rat #221	Sucrose	Femoral	240	2.13
Rat #221	Sucrose	Femoral	360	2.29
Rat #222	Sucrose	Femoral	0	2.25
Rat #222	Sucrose	Femoral	15	2.87
Rat #222	Sucrose	Femoral	30	5.92
Rat #222	Sucrose	Femoral	60	4.84
Rat #222	Sucrose	Femoral	120	2.50
Rat #222	Sucrose	Femoral	240	2.54
Rat #222	Sucrose	Femoral	360	2.27
Rat #228	Sucrose	Femoral	0	1.66
Rat #228	Sucrose	Femoral	15	4.30
Rat #228	Sucrose	Femoral	30	4.37
Rat #228	Sucrose	Femoral	60	3.46
Rat #228	Sucrose	Femoral	120	2.19
Rat #228	Sucrose	Femoral	240	1.88
Rat #228	Sucrose	Femoral	360	1.68
Rat #231	Sucrose	Femoral	0	1.45
Rat #231	Sucrose	Femoral	15	4.52
Rat #231	Sucrose	Femoral	30	5.62
Rat #231	Sucrose	Femoral	60	4.40
Rat #231	Sucrose	Femoral	120	1.93
Rat #231	Sucrose	Femoral	240	2.11
Rat #231	Sucrose	Femoral	360	1.59
Rat #236	Sucrose	Femoral	0	1.15
Rat #236	Sucrose	Femoral	15	4.57
Rat #236	Sucrose	Femoral	30	5.31
Rat #236	Sucrose	Femoral	60	3.15
Rat #236	Sucrose	Femoral	120	1.53
Rat #236	Sucrose	Femoral	240	2.16
Rat #236	Sucrose	Femoral	360	1.86
Rat #237	Sucrose	Femoral	0	1.63
Rat #237	Sucrose	Femoral	15	6.30
Rat #237	Sucrose	Femoral	30	6.73
Rat #237	Sucrose	Femoral	60	3.55
Rat #237	Sucrose	Femoral	120	2.36
Rat #237	Sucrose	Femoral	240	2.00
Rat #237	Sucrose	Femoral	360	2.03
Rat #101	Glucose	Femoral	0	1.53
Rat #101	Glucose	Femoral	15	1.57
Rat #101	Glucose	Femoral	30	1.59
Rat #101	Glucose	Femoral	60	1.64
Rat #101	Glucose	Femoral	120	1.66
Rat #101	Glucose	Femoral	240	1.70
Rat #101	Glucose	Femoral	360	1.45
Rat #103	Glucose	Femoral	0	1.40
Rat #103	Glucose	Femoral	15	1.49

S1 Table. Data for Fructose Concentration versus Time in Femoral and Portal Veins.

Rat ID	Gavage Treatment	Vein	Time (min)	Fructose (mg/dL)
Rat #103	Glucose	Femoral	30	1.45
Rat #103	Glucose	Femoral	60	1.50
Rat #103	Glucose	Femoral	120	1.63
Rat #103	Glucose	Femoral	240	1.59
Rat #103	Glucose	Femoral	360	1.51
Rat #107	Glucose	Femoral	0	1.68
Rat #107	Glucose	Femoral	15	1.95
Rat #107	Glucose	Femoral	30	1.78
Rat #107	Glucose	Femoral	60	1.81
Rat #107	Glucose	Femoral	120	2.02
Rat #107	Glucose	Femoral	240	1.80
Rat #107	Glucose	Femoral	360	1.79
Rat #110	Glucose	Femoral	0	1.59
Rat #110	Glucose	Femoral	15	1.87
Rat #110	Glucose	Femoral	30	1.81
Rat #110	Glucose	Femoral	60	1.95
Rat #110	Glucose	Femoral	120	1.86
Rat #110	Glucose	Femoral	240	1.93
Rat #110	Glucose	Femoral	360	1.79
Rat #115	Glucose	Femoral	0	1.67
Rat #115	Glucose	Femoral	15	1.89
Rat #115	Glucose	Femoral	30	1.77
Rat #115	Glucose	Femoral	60	1.94
Rat #115	Glucose	Femoral	120	1.82
Rat #115	Glucose	Femoral	240	1.67
Rat #115	Glucose	Femoral	360	1.76
Rat #118	Glucose	Femoral	0	1.66
Rat #118	Glucose	Femoral	15	1.95
Rat #118	Glucose	Femoral	30	2.25
Rat #118	Glucose	Femoral	60	2.30
Rat #118	Glucose	Femoral	120	2.17
Rat #118	Glucose	Femoral	240	2.12
Rat #118	Glucose	Femoral	360	2.01
Rat #122	Glucose	Femoral	0	1.56
Rat #122	Glucose	Femoral	15	1.66
Rat #122	Glucose	Femoral	30	1.62
Rat #122	Glucose	Femoral	60	1.71
Rat #122	Glucose	Femoral	120	1.63
Rat #122	Glucose	Femoral	240	1.65
Rat #122	Glucose	Femoral	360	1.71
Rat #124	Glucose	Femoral	0	1.79
Rat #124	Glucose	Femoral	15	1.94
Rat #124	Glucose	Femoral	30	1.96
Rat #124	Glucose	Femoral	60	1.94
Rat #124	Glucose	Femoral	120	1.77
Rat #124	Glucose	Femoral	240	1.70
Rat #124	Glucose	Femoral	360	1.94
Rat #104	Fructose	Femoral	0	1.57
Rat #104	Fructose	Femoral	15	5.98
Rat #104	Fructose	Femoral	30	6.55
Rat #104	Fructose	Femoral	60	3.73
Rat #104	Fructose	Femoral	120	3.34
Rat #104	Fructose	Femoral	240	1.78
Rat #104	Fructose	Femoral	360	1.71
Rat #106	Fructose	Femoral	0	1.26
Rat #106	Fructose	Femoral	15	7.82
Rat #106	Fructose	Femoral	30	8.13
Rat #106	Fructose	Femoral	60	6.22
Rat #106	Fructose	Femoral	120	3.00
Rat #106	Fructose	Femoral	240	1.96
Rat #106	Fructose	Femoral	360	1.89
Rat #109	Fructose	Femoral	0	1.63
Rat #109	Fructose	Femoral	15	5.99
Rat #109	Fructose	Femoral	30	8.39

S1 Table. Data for Fructose Concentration versus Time in Femoral and Portal Veins.

Rat ID	Gavage Treatment	Vein	Time (min)	Fructose (mg/dL)
Rat #109	Fructose	Femoral	60	5.03
Rat #109	Fructose	Femoral	120	2.90
Rat #109	Fructose	Femoral	240	2.02
Rat #109	Fructose	Femoral	360	1.99
Rat #111	Fructose	Femoral	0	1.74
Rat #111	Fructose	Femoral	15	6.18
Rat #111	Fructose	Femoral	30	7.88
Rat #111	Fructose	Femoral	60	6.37
Rat #111	Fructose	Femoral	120	3.21
Rat #111	Fructose	Femoral	240	1.96
Rat #111	Fructose	Femoral	360	2.08
Rat #113	Fructose	Femoral	0	1.84
Rat #113	Fructose	Femoral	15	5.66
Rat #113	Fructose	Femoral	30	6.62
Rat #113	Fructose	Femoral	60	4.93
Rat #113	Fructose	Femoral	120	3.60
Rat #113	Fructose	Femoral	240	1.82
Rat #113	Fructose	Femoral	360	1.69
Rat #116	Fructose	Femoral	0	1.99
Rat #116	Fructose	Femoral	15	6.61
Rat #116	Fructose	Femoral	30	8.45
Rat #116	Fructose	Femoral	60	3.46
Rat #116	Fructose	Femoral	120	2.92
Rat #116	Fructose	Femoral	240	1.89
Rat #116	Fructose	Femoral	360	2.22
Rat #119	Fructose	Femoral	0	1.95
Rat #119	Fructose	Femoral	15	6.05
Rat #119	Fructose	Femoral	30	7.03
Rat #119	Fructose	Femoral	60	7.18
Rat #119	Fructose	Femoral	120	3.06
Rat #119	Fructose	Femoral	240	1.88
Rat #119	Fructose	Femoral	360	1.90
Rat #121	Fructose	Femoral	0	1.61
Rat #121	Fructose	Femoral	15	5.51
Rat #121	Fructose	Femoral	30	6.56
Rat #121	Fructose	Femoral	60	6.09
Rat #121	Fructose	Femoral	120	3.54
Rat #121	Fructose	Femoral	240	1.82
Rat #121	Fructose	Femoral	360	1.66
Rat #201	45/55 Glucose/Fructose	Femoral	0	1.84
Rat #201	45/55 Glucose/Fructose	Femoral	15	4.95
Rat #201	45/55 Glucose/Fructose	Femoral	30	5.74
Rat #201	45/55 Glucose/Fructose	Femoral	60	2.95
Rat #201	45/55 Glucose/Fructose	Femoral	120	1.57
Rat #201	45/55 Glucose/Fructose	Femoral	240	1.43
Rat #201	45/55 Glucose/Fructose	Femoral	360	1.29
Rat #203	45/55 Glucose/Fructose	Femoral	0	1.33
Rat #203	45/55 Glucose/Fructose	Femoral	15	5.52
Rat #203	45/55 Glucose/Fructose	Femoral	30	6.21
Rat #203	45/55 Glucose/Fructose	Femoral	60	2.52
Rat #203	45/55 Glucose/Fructose	Femoral	120	2.09
Rat #203	45/55 Glucose/Fructose	Femoral	240	1.76
Rat #203	45/55 Glucose/Fructose	Femoral	360	1.73
Rat #212	45/55 Glucose/Fructose	Femoral	0	1.48
Rat #212	45/55 Glucose/Fructose	Femoral	15	4.38
Rat #212	45/55 Glucose/Fructose	Femoral	30	6.35
Rat #212	45/55 Glucose/Fructose	Femoral	60	3.51
Rat #212	45/55 Glucose/Fructose	Femoral	120	1.73
Rat #212	45/55 Glucose/Fructose	Femoral	240	1.31
Rat #212	45/55 Glucose/Fructose	Femoral	360	1.51
Rat #216	45/55 Glucose/Fructose	Femoral	0	1.10
Rat #216	45/55 Glucose/Fructose	Femoral	15	4.74
Rat #216	45/55 Glucose/Fructose	Femoral	30	5.32
Rat #216	45/55 Glucose/Fructose	Femoral	60	4.18

S1 Table. Data for Fructose Concentration versus Time in Femoral and Portal Veins.

Rat ID	Gavage Treatment	Vein	Time (min)	Fructose (mg/dL)
Rat #216	45/55 Glucose/Fructose	Femoral	120	2.31
Rat #216	45/55 Glucose/Fructose	Femoral	240	2.40
Rat #216	45/55 Glucose/Fructose	Femoral	360	1.95
Rat #219	45/55 Glucose/Fructose	Femoral	0	1.60
Rat #219	45/55 Glucose/Fructose	Femoral	15	5.70
Rat #219	45/55 Glucose/Fructose	Femoral	30	5.67
Rat #219	45/55 Glucose/Fructose	Femoral	60	3.85
Rat #219	45/55 Glucose/Fructose	Femoral	120	2.30
Rat #219	45/55 Glucose/Fructose	Femoral	240	1.88
Rat #219	45/55 Glucose/Fructose	Femoral	360	1.45
Rat #223	45/55 Glucose/Fructose	Femoral	0	1.83
Rat #223	45/55 Glucose/Fructose	Femoral	15	5.79
Rat #223	45/55 Glucose/Fructose	Femoral	30	6.63
Rat #223	45/55 Glucose/Fructose	Femoral	60	3.79
Rat #223	45/55 Glucose/Fructose	Femoral	120	2.72
Rat #223	45/55 Glucose/Fructose	Femoral	240	1.93
Rat #223	45/55 Glucose/Fructose	Femoral	360	1.60
Rat #226	45/55 Glucose/Fructose	Femoral	0	1.66
Rat #226	45/55 Glucose/Fructose	Femoral	15	5.86
Rat #226	45/55 Glucose/Fructose	Femoral	30	6.69
Rat #226	45/55 Glucose/Fructose	Femoral	60	3.71
Rat #226	45/55 Glucose/Fructose	Femoral	120	1.98
Rat #226	45/55 Glucose/Fructose	Femoral	240	1.88
Rat #226	45/55 Glucose/Fructose	Femoral	360	1.41
Rat #230	45/55 Glucose/Fructose	Femoral	0	1.63
Rat #230	45/55 Glucose/Fructose	Femoral	15	6.21
Rat #230	45/55 Glucose/Fructose	Femoral	30	7.19
Rat #230	45/55 Glucose/Fructose	Femoral	60	3.87
Rat #230	45/55 Glucose/Fructose	Femoral	120	2.35
Rat #230	45/55 Glucose/Fructose	Femoral	240	2.07
Rat #230	45/55 Glucose/Fructose	Femoral	360	2.19
Rat #233	45/55 Glucose/Fructose	Femoral	0	1.19
Rat #233	45/55 Glucose/Fructose	Femoral	15	6.41
Rat #233	45/55 Glucose/Fructose	Femoral	30	6.25
Rat #233	45/55 Glucose/Fructose	Femoral	60	2.98
Rat #233	45/55 Glucose/Fructose	Femoral	120	2.00
Rat #233	45/55 Glucose/Fructose	Femoral	240	2.27
Rat #233	45/55 Glucose/Fructose	Femoral	360	2.22
Rat #239	45/55 Glucose/Fructose	Femoral	0	2.00
Rat #239	45/55 Glucose/Fructose	Femoral	15	8.13
Rat #239	45/55 Glucose/Fructose	Femoral	30	7.81
Rat #239	45/55 Glucose/Fructose	Femoral	60	7.91
Rat #239	45/55 Glucose/Fructose	Femoral	120	2.75
Rat #239	45/55 Glucose/Fructose	Femoral	240	2.28
Rat #239	45/55 Glucose/Fructose	Femoral	360	1.76
Rat #102	Water	Portal	0	1.45
Rat #102	Water	Portal	15	1.34
Rat #102	Water	Portal	30	1.73
Rat #102	Water	Portal	60	1.81
Rat #102	Water	Portal	120	1.82
Rat #102	Water	Portal	240	1.88
Rat #102	Water	Portal	360	1.66
Rat #105	Water	Portal	0	1.73
Rat #105	Water	Portal	15	1.81
Rat #105	Water	Portal	30	2.01
Rat #105	Water	Portal	60	1.76
Rat #105	Water	Portal	120	1.90
Rat #105	Water	Portal	240	2.01
Rat #105	Water	Portal	360	1.77
Rat #108	Water	Portal	0	1.59
Rat #108	Water	Portal	15	1.72
Rat #108	Water	Portal	30	1.95
Rat #108	Water	Portal	60	1.82
Rat #108	Water	Portal	120	2.02

S1 Table. Data for Fructose Concentration versus Time in Femoral and Portal Veins.

Rat ID	Gavage Treatment	Vein	Time (min)	Fructose (mg/dL)
Rat #108	Water	Portal	240	2.00
Rat #108	Water	Portal	360	1.97
Rat #112	Water	Portal	0	1.80
Rat #112	Water	Portal	15	1.82
Rat #112	Water	Portal	30	1.91
Rat #112	Water	Portal	60	1.88
Rat #112	Water	Portal	120	1.78
Rat #112	Water	Portal	240	1.86
Rat #112	Water	Portal	360	1.76
Rat #114	Water	Portal	0	1.62
Rat #114	Water	Portal	15	1.72
Rat #114	Water	Portal	30	1.65
Rat #114	Water	Portal	60	1.71
Rat #114	Water	Portal	120	1.72
Rat #114	Water	Portal	240	1.66
Rat #114	Water	Portal	360	1.72
Rat #117	Water	Portal	0	1.87
Rat #117	Water	Portal	15	2.13
Rat #117	Water	Portal	30	2.09
Rat #117	Water	Portal	60	2.14
Rat #117	Water	Portal	120	1.98
Rat #117	Water	Portal	240	2.19
Rat #117	Water	Portal	360	2.04
Rat #120	Water	Portal	0	1.76
Rat #120	Water	Portal	15	1.82
Rat #120	Water	Portal	30	1.75
Rat #120	Water	Portal	60	1.76
Rat #120	Water	Portal	120	1.86
Rat #120	Water	Portal	240	1.95
Rat #120	Water	Portal	360	1.81
Rat #123	Water	Portal	0	1.72
Rat #123	Water	Portal	15	1.66
Rat #123	Water	Portal	30	1.65
Rat #123	Water	Portal	60	1.72
Rat #123	Water	Portal	120	1.73
Rat #123	Water	Portal	240	1.79
Rat #123	Water	Portal	360	1.63
Rat #204	Sucrose	Portal	0	0.86
Rat #204	Sucrose	Portal	15	51.34
Rat #204	Sucrose	Portal	30	13.83
Rat #204	Sucrose	Portal	60	15.23
Rat #204	Sucrose	Portal	120	1.77
Rat #204	Sucrose	Portal	240	1.10
Rat #204	Sucrose	Portal	360	1.04
Rat #205	Sucrose	Portal	0	1.15
Rat #205	Sucrose	Portal	15	26.04
Rat #205	Sucrose	Portal	30	26.03
Rat #205	Sucrose	Portal	60	4.71
Rat #205	Sucrose	Portal	120	1.19
Rat #205	Sucrose	Portal	240	1.02
Rat #205	Sucrose	Portal	360	1.34
Rat #211	Sucrose	Portal	0	1.23
Rat #211	Sucrose	Portal	15	42.39
Rat #211	Sucrose	Portal	30	40.87
Rat #211	Sucrose	Portal	60	9.73
Rat #211	Sucrose	Portal	120	5.80
Rat #211	Sucrose	Portal	240	1.05
Rat #211	Sucrose	Portal	360	1.55
Rat #221	Sucrose	Portal	0	1.67
Rat #221	Sucrose	Portal	15	28.09
Rat #221	Sucrose	Portal	30	38.59
Rat #221	Sucrose	Portal	60	10.48
Rat #221	Sucrose	Portal	120	2.65
Rat #221	Sucrose	Portal	240	1.85

S1 Table. Data for Fructose Concentration versus Time in Femoral and Portal Veins.

Rat ID	Gavage Treatment	Vein	Time (min)	Fructose (mg/dL)
Rat #221	Sucrose	Portal	360	2.14
Rat #222	Sucrose	Portal	0	1.66
Rat #222	Sucrose	Portal	15	12.89
Rat #222	Sucrose	Portal	30	34.32
Rat #222	Sucrose	Portal	60	13.42
Rat #222	Sucrose	Portal	120	2.95
Rat #222	Sucrose	Portal	240	2.24
Rat #222	Sucrose	Portal	360	1.53
Rat #228	Sucrose	Portal	0	1.76
Rat #228	Sucrose	Portal	15	25.79
Rat #228	Sucrose	Portal	30	27.38
Rat #228	Sucrose	Portal	60	12.10
Rat #228	Sucrose	Portal	120	3.09
Rat #228	Sucrose	Portal	240	2.05
Rat #228	Sucrose	Portal	360	1.59
Rat #231	Sucrose	Portal	0	1.30
Rat #231	Sucrose	Portal	15	22.70
Rat #231	Sucrose	Portal	30	23.40
Rat #231	Sucrose	Portal	60	12.98
Rat #231	Sucrose	Portal	120	2.12
Rat #231	Sucrose	Portal	240	1.68
Rat #231	Sucrose	Portal	360	1.41
Rat #236	Sucrose	Portal	0	1.22
Rat #236	Sucrose	Portal	15	18.27
Rat #236	Sucrose	Portal	30	32.66
Rat #236	Sucrose	Portal	60	6.98
Rat #236	Sucrose	Portal	120	1.87
Rat #236	Sucrose	Portal	240	1.39
Rat #236	Sucrose	Portal	360	1.22
Rat #237	Sucrose	Portal	0	1.77
Rat #237	Sucrose	Portal	15	37.04
Rat #237	Sucrose	Portal	30	24.14
Rat #237	Sucrose	Portal	60	4.48
Rat #237	Sucrose	Portal	120	3.52
Rat #237	Sucrose	Portal	240	1.66
Rat #237	Sucrose	Portal	360	1.78
Rat #101	Glucose	Portal	0	1.54
Rat #101	Glucose	Portal	15	1.65
Rat #101	Glucose	Portal	30	1.90
Rat #101	Glucose	Portal	60	1.62
Rat #101	Glucose	Portal	120	1.65
Rat #101	Glucose	Portal	240	1.67
Rat #101	Glucose	Portal	360	1.50
Rat #103	Glucose	Portal	0	1.43
Rat #103	Glucose	Portal	15	1.59
Rat #103	Glucose	Portal	30	1.51
Rat #103	Glucose	Portal	60	1.49
Rat #103	Glucose	Portal	120	1.48
Rat #103	Glucose	Portal	240	1.67
Rat #103	Glucose	Portal	360	1.56
Rat #107	Glucose	Portal	0	1.54
Rat #107	Glucose	Portal	15	1.99
Rat #107	Glucose	Portal	30	1.88
Rat #107	Glucose	Portal	60	1.83
Rat #107	Glucose	Portal	120	1.76
Rat #107	Glucose	Portal	240	1.96
Rat #107	Glucose	Portal	360	1.72
Rat #110	Glucose	Portal	0	1.74
Rat #110	Glucose	Portal	15	1.90
Rat #110	Glucose	Portal	30	1.84
Rat #110	Glucose	Portal	60	1.82
Rat #110	Glucose	Portal	120	1.87
Rat #110	Glucose	Portal	240	1.76
Rat #110	Glucose	Portal	360	1.83

S1 Table. Data for Fructose Concentration versus Time in Femoral and Portal Veins.

Rat ID	Gavage Treatment	Vein	Time (min)	Fructose (mg/dL)
Rat #115	Glucose	Portal	0	1.85
Rat #115	Glucose	Portal	15	1.89
Rat #115	Glucose	Portal	30	1.87
Rat #115	Glucose	Portal	60	1.81
Rat #115	Glucose	Portal	120	1.88
Rat #115	Glucose	Portal	240	1.93
Rat #115	Glucose	Portal	360	1.87
Rat #118	Glucose	Portal	0	1.74
Rat #118	Glucose	Portal	15	2.38
Rat #118	Glucose	Portal	30	2.26
Rat #118	Glucose	Portal	60	2.15
Rat #118	Glucose	Portal	120	2.09
Rat #118	Glucose	Portal	240	2.12
Rat #118	Glucose	Portal	360	2.09
Rat #122	Glucose	Portal	0	1.65
Rat #122	Glucose	Portal	15	1.91
Rat #122	Glucose	Portal	30	1.69
Rat #122	Glucose	Portal	60	1.61
Rat #122	Glucose	Portal	120	1.66
Rat #122	Glucose	Portal	240	1.54
Rat #122	Glucose	Portal	360	1.64
Rat #124	Glucose	Portal	0	1.81
Rat #124	Glucose	Portal	15	1.92
Rat #124	Glucose	Portal	30	1.73
Rat #124	Glucose	Portal	60	1.94
Rat #124	Glucose	Portal	120	1.99
Rat #124	Glucose	Portal	240	1.88
Rat #124	Glucose	Portal	360	1.82
Rat #104	Fructose	Portal	0	1.58
Rat #104	Fructose	Portal	15	40.59
Rat #104	Fructose	Portal	30	31.20
Rat #104	Fructose	Portal	60	27.13
Rat #104	Fructose	Portal	120	10.36
Rat #104	Fructose	Portal	240	2.02
Rat #104	Fructose	Portal	360	1.47
Rat #106	Fructose	Portal	0	2.55
Rat #106	Fructose	Portal	15	39.19
Rat #106	Fructose	Portal	30	30.02
Rat #106	Fructose	Portal	60	22.41
Rat #106	Fructose	Portal	120	5.99
Rat #106	Fructose	Portal	240	2.07
Rat #106	Fructose	Portal	360	1.83
Rat #109	Fructose	Portal	0	1.74
Rat #109	Fructose	Portal	15	38.46
Rat #109	Fructose	Portal	30	29.26
Rat #109	Fructose	Portal	60	18.64
Rat #109	Fructose	Portal	120	8.26
Rat #109	Fructose	Portal	240	1.82
Rat #109	Fructose	Portal	360	1.72
Rat #111	Fructose	Portal	0	1.73
Rat #111	Fructose	Portal	15	52.32
Rat #111	Fructose	Portal	30	60.21
Rat #111	Fructose	Portal	60	49.43
Rat #111	Fructose	Portal	120	17.84
Rat #111	Fructose	Portal	240	1.89
Rat #111	Fructose	Portal	360	0.72
Rat #113	Fructose	Portal	0	1.47
Rat #113	Fructose	Portal	15	59.19
Rat #113	Fructose	Portal	30	43.41
Rat #113	Fructose	Portal	60	24.25
Rat #113	Fructose	Portal	120	12.87
Rat #113	Fructose	Portal	240	1.98
Rat #113	Fructose	Portal	360	1.48
Rat #116	Fructose	Portal	0	1.81

S1 Table. Data for Fructose Concentration versus Time in Femoral and Portal Veins.

Rat ID	Gavage Treatment	Vein	Time (min)	Fructose (mg/dL)
Rat #116	Fructose	Portal	15	37.50
Rat #116	Fructose	Portal	30	30.73
Rat #116	Fructose	Portal	60	7.43
Rat #116	Fructose	Portal	120	2.94
Rat #116	Fructose	Portal	240	1.91
Rat #116	Fructose	Portal	360	2.23
Rat #119	Fructose	Portal	0	1.80
Rat #119	Fructose	Portal	15	38.62
Rat #119	Fructose	Portal	30	37.79
Rat #119	Fructose	Portal	60	31.85
Rat #119	Fructose	Portal	120	5.41
Rat #119	Fructose	Portal	240	2.02
Rat #119	Fructose	Portal	360	1.94
Rat #121	Fructose	Portal	0	1.60
Rat #121	Fructose	Portal	15	25.16
Rat #121	Fructose	Portal	30	27.36
Rat #121	Fructose	Portal	60	29.61
Rat #121	Fructose	Portal	120	13.62
Rat #121	Fructose	Portal	240	1.76
Rat #121	Fructose	Portal	360	1.71
Rat #201	45/55 Glucose/Fructose	Portal	0	0.85
Rat #201	45/55 Glucose/Fructose	Portal	15	28.58
Rat #201	45/55 Glucose/Fructose	Portal	30	35.35
Rat #201	45/55 Glucose/Fructose	Portal	60	11.32
Rat #201	45/55 Glucose/Fructose	Portal	120	1.23
Rat #201	45/55 Glucose/Fructose	Portal	240	1.18
Rat #201	45/55 Glucose/Fructose	Portal	360	1.07
Rat #203	45/55 Glucose/Fructose	Portal	0	0.85
Rat #203	45/55 Glucose/Fructose	Portal	15	50.91
Rat #203	45/55 Glucose/Fructose	Portal	30	40.43
Rat #203	45/55 Glucose/Fructose	Portal	60	10.03
Rat #203	45/55 Glucose/Fructose	Portal	120	1.96
Rat #203	45/55 Glucose/Fructose	Portal	240	1.47
Rat #203	45/55 Glucose/Fructose	Portal	360	1.63
Rat #212	45/55 Glucose/Fructose	Portal	0	1.17
Rat #212	45/55 Glucose/Fructose	Portal	15	25.61
Rat #212	45/55 Glucose/Fructose	Portal	30	37.76
Rat #212	45/55 Glucose/Fructose	Portal	60	11.15
Rat #212	45/55 Glucose/Fructose	Portal	120	2.01
Rat #212	45/55 Glucose/Fructose	Portal	240	0.99
Rat #212	45/55 Glucose/Fructose	Portal	360	1.02
Rat #216	45/55 Glucose/Fructose	Portal	0	0.68
Rat #216	45/55 Glucose/Fructose	Portal	15	24.20
Rat #216	45/55 Glucose/Fructose	Portal	30	21.28
Rat #216	45/55 Glucose/Fructose	Portal	60	18.23
Rat #216	45/55 Glucose/Fructose	Portal	120	4.53
Rat #216	45/55 Glucose/Fructose	Portal	240	2.17
Rat #216	45/55 Glucose/Fructose	Portal	360	0.99
Rat #219	45/55 Glucose/Fructose	Portal	0	1.75
Rat #219	45/55 Glucose/Fructose	Portal	15	26.15
Rat #219	45/55 Glucose/Fructose	Portal	30	28.00
Rat #219	45/55 Glucose/Fructose	Portal	60	7.55
Rat #219	45/55 Glucose/Fructose	Portal	120	2.44
Rat #219	45/55 Glucose/Fructose	Portal	240	1.92
Rat #219	45/55 Glucose/Fructose	Portal	360	1.75
Rat #223	45/55 Glucose/Fructose	Portal	0	1.81
Rat #223	45/55 Glucose/Fructose	Portal	15	31.71
Rat #223	45/55 Glucose/Fructose	Portal	30	19.17
Rat #223	45/55 Glucose/Fructose	Portal	60	13.89
Rat #223	45/55 Glucose/Fructose	Portal	120	4.33
Rat #223	45/55 Glucose/Fructose	Portal	240	1.75
Rat #223	45/55 Glucose/Fructose	Portal	360	1.84
Rat #226	45/55 Glucose/Fructose	Portal	0	1.64
Rat #226	45/55 Glucose/Fructose	Portal	15	36.54

S1 Table. Data for Fructose Concentration versus Time in Femoral and Portal Veins.

Rat ID	Gavage Treatment	Vein	Time (min)	Fructose (mg/dL)
Rat #226	45/55 Glucose/Fructose	Portal	30	43.78
Rat #226	45/55 Glucose/Fructose	Portal	60	6.42
Rat #226	45/55 Glucose/Fructose	Portal	120	2.08
Rat #226	45/55 Glucose/Fructose	Portal	240	1.83
Rat #226	45/55 Glucose/Fructose	Portal	360	1.86
Rat #230	45/55 Glucose/Fructose	Portal	0	1.32
Rat #230	45/55 Glucose/Fructose	Portal	15	28.51
Rat #230	45/55 Glucose/Fructose	Portal	30	35.17
Rat #230	45/55 Glucose/Fructose	Portal	60	7.61
Rat #230	45/55 Glucose/Fructose	Portal	120	2.58
Rat #230	45/55 Glucose/Fructose	Portal	240	2.10
Rat #230	45/55 Glucose/Fructose	Portal	360	2.13
Rat #233	45/55 Glucose/Fructose	Portal	0	1.05
Rat #233	45/55 Glucose/Fructose	Portal	15	30.35
Rat #233	45/55 Glucose/Fructose	Portal	30	28.99
Rat #233	45/55 Glucose/Fructose	Portal	60	8.64
Rat #233	45/55 Glucose/Fructose	Portal	120	1.37
Rat #233	45/55 Glucose/Fructose	Portal	240	1.64
Rat #233	45/55 Glucose/Fructose	Portal	360	1.70
Rat #239	45/55 Glucose/Fructose	Portal	0	1.91
Rat #239	45/55 Glucose/Fructose	Portal	15	41.17
Rat #239	45/55 Glucose/Fructose	Portal	30	23.85
Rat #239	45/55 Glucose/Fructose	Portal	60	9.11
Rat #239	45/55 Glucose/Fructose	Portal	120	4.59
Rat #239	45/55 Glucose/Fructose	Portal	240	1.49
Rat #239	45/55 Glucose/Fructose	Portal	360	2.04
