# Supplementary material for: Effects of fructose-containing sweeteners on fructose intestinal, hepatic, and oral bioavailability in dual-catheterized rats
Source: PLoS One. 2018 Nov 8;13(11):e0207024. doi: 10.1371/journal.pone.0207024 (PMC6224110; doi:10.1371/journal.pone.0207024)
Supplement: S2 Table — AUC = area under the curve. PV: portal vein. SYS: femoral vein. Tmax = time at maximum observed concentration. (PDF) [file pone.0207024.s002.pdf]

**S2 Table. Data for Fructose AUC and $T_{max}$ in Femoral and Portal Veins.**

| Rat ID | Gavage Treatment | Fructose $AUC_{sys}$ (min*mg/dl) | Fructose $AUC_{pv}$ (min*mg/dl) | Fructose $T_{max\_sys}$ (min) | Fructose $T_{max\_pv}$ (min) |
|---|---|---|---|---|---|
| Rat #102 | Water | 633.66 | 638.62 | 238 | 238 |
| Rat #105 | Water | 651.15 | 682.44 | 57 | 35 |
| Rat #108 | Water | 709.25 | 702.76 | 57 | 119 |
| Rat #112 | Water | 674.64 | 656.86 | 243 | 35 |
| Rat #114 | Water | 619.48 | 608.54 | 60 | 18 |
| Rat #117 | Water | 715.94 | 752.89 | 119 | 240 |
| Rat #120 | Water | 695.77 | 670.16 | 239 | 238 |
| Rat #123 | Water | 589.25 | 620.13 | 63 | 240 |
| Rat #204 | Sucrose | 676.08 | 1899.13 | 16 | 16 |
| Rat #205 | Sucrose | 759.67 | 1462.61 | 33 | 13 |
| Rat #211 | Sucrose | 657.32 | 2645.48 | 30 | 19 |
| Rat #221 | Sucrose | 1025.93 | 2222.06 | 33 | 33 |
| Rat #222 | Sucrose | 1063.43 | 2060.87 | 35 | 35 |
| Rat #228 | Sucrose | 846.59 | 2059.07 | 30 | 30 |
| Rat #231 | Sucrose | 913.45 | 1853.03 | 32 | 31 |
| Rat #236 | Sucrose | 835.85 | 1622.95 | 30 | 30 |
| Rat #237 | Sucrose | 977.41 | 1829.86 | 30 | 18 |
| Rat #101 | Glucose | 584.07 | 589.51 | 236 | 36 |
| Rat #103 | Glucose | 559.99 | 562.66 | 118 | 237 |
| Rat #107 | Glucose | 665.87 | 662.97 | 117 | 22 |
| Rat #110 | Glucose | 675.36 | 654.84 | 60 | 12 |
| Rat #115 | Glucose | 636.92 | 678.68 | 59 | 238 |
| Rat #118 | Glucose | 765.76 | 764.24 | 63 | 18 |
| Rat #122 | Glucose | 596.80 | 583.02 | 65 | 16 |
| Rat #124 | Glucose | 653.07 | 681.60 | 29 | 118 |
| Rat #104 | Fructose | 1020.07 | 3606.07 | 29 | 19 |
| Rat #106 | Fructose | 1212.87 | 3150.08 | 31 | 15 |
| Rat #109 | Fructose | 1128.27 | 3019.54 | 29 | 15 |
| Rat #111 | Fructose | 1214.93 | 5887.92 | 27 | 27 |
| Rat #113 | Fructose | 1122.90 | 4412.84 | 28 | 17 |
| Rat #116 | Fructose | 1106.06 | 2312.68 | 30 | 15 |
| Rat #119 | Fructose | 1173.68 | 3436.79 | 58 | 13 |
| Rat #121 | Fructose | 1130.55 | 3552.01 | 30 | 58 |
| Rat #201 | 45/55 Glucose/Fructose | 726.62 | 1845.62 | 30 | 30 |
| Rat #203 | 45/55 Glucose/Fructose | 836.07 | 2325.78 | 29 | 17 |
| Rat #212 | 45/55 Glucose/Fructose | 773.94 | 1967.70 | 32 | 31 |
| Rat #216 | 45/55 Glucose/Fructose | 992.69 | 2285.21 | 31 | 14 |
| Rat #219 | 45/55 Glucose/Fructose | 908.82 | 1858.65 | 14 | 33 |
| Rat #223 | 45/55 Glucose/Fructose | 985.84 | 2179.64 | 32 | 14 |
| Rat #226 | 45/55 Glucose/Fructose | 903.38 | 2197.72 | 30 | 30 |
| Rat #230 | 45/55 Glucose/Fructose | 1033.30 | 2095.44 | 28 | 28 |
| Rat #233 | 45/55 Glucose/Fructose | 967.34 | 1877.28 | 14 | 13 |
| Rat #239 | 45/55 Glucose/Fructose | 1263.17 | 2238.63 | 14 | 13 |

AUC = area under the curve. PV: portal vein. SYS: femoral vein. $T_{max}$ = time at maximum observed concentration.
