# Supplementary material for: Effects of fructose-containing sweeteners on fructose intestinal, hepatic, and oral bioavailability in dual-catheterized rats
Source: PLoS One. 2018 Nov 8;13(11):e0207024. doi: 10.1371/journal.pone.0207024 (PMC6224110; doi:10.1371/journal.pone.0207024)
Supplement: S3 Table — AdjCmax = maximum observed concentration—concentration at time = 0. Cmax = maximum observed concentration. (PDF) [file pone.0207024.s003.pdf]

S3 Table. Data for Fructose AdjC_{max} in Femoral and Portal Veins.

Rat ID	Gavage Treatment	Vein	Fructose C_{max} (mg/dL)	Fructose Baseline (Time = 0) (mg/dL)	Fructose AdjC_{max} (mg/dL)
Rat #102	Water	Femoral	1.87	1.47	0.39
Rat #105	Water	Femoral	1.98	1.69	0.29
Rat #108	Water	Femoral	2.06	1.61	0.46
Rat #112	Water	Femoral	1.93	1.70	0.23
Rat #114	Water	Femoral	1.79	1.68	0.11
Rat #117	Water	Femoral	2.23	1.98	0.26
Rat #120	Water	Femoral	1.98	1.90	0.08
Rat #123	Water	Femoral	1.68	1.57	0.10
Rat #204	Sucrose	Femoral	4.44	1.32	3.12
Rat #205	Sucrose	Femoral	6.52	1.86	4.66
Rat #211	Sucrose	Femoral	4.36	1.36	3.00
Rat #221	Sucrose	Femoral	6.89	1.84	5.05
Rat #222	Sucrose	Femoral	5.92	2.25	3.67
Rat #228	Sucrose	Femoral	4.37	1.66	2.71
Rat #231	Sucrose	Femoral	5.62	1.45	4.17
Rat #236	Sucrose	Femoral	5.31	1.15	4.16
Rat #237	Sucrose	Femoral	6.73	1.63	5.11
Rat #101	Glucose	Femoral	1.70	1.53	0.16
Rat #103	Glucose	Femoral	1.63	1.40	0.23
Rat #107	Glucose	Femoral	2.02	1.68	0.33
Rat #110	Glucose	Femoral	1.95	1.59	0.36
Rat #115	Glucose	Femoral	1.94	1.67	0.27
Rat #118	Glucose	Femoral	2.30	1.66	0.64
Rat #122	Glucose	Femoral	1.71	1.56	0.15
Rat #124	Glucose	Femoral	1.96	1.79	0.17
Rat #104	Fructose	Femoral	6.55	1.57	4.98
Rat #106	Fructose	Femoral	8.13	1.26	6.87
Rat #109	Fructose	Femoral	8.39	1.63	6.76
Rat #111	Fructose	Femoral	7.88	1.74	6.14
Rat #113	Fructose	Femoral	6.62	1.84	4.78
Rat #116	Fructose	Femoral	8.45	1.99	6.46
Rat #119	Fructose	Femoral	7.18	1.95	5.23
Rat #121	Fructose	Femoral	6.56	1.61	4.95
Rat #201	45/55 Glucose/Fructose	Femoral	5.74	1.84	3.90

S3 Table. Data for Fructose AdjC$_{max}$ in Femoral and Portal Veins.

Rat ID	Gavage Treatment	Vein	Fructose C_{max} (mg/dL)	Fructose Baseline (Time = 0) (mg/dL)	Fructose AdjC_{max} (mg/dL)
Rat #203	45/55 Glucose/Fructose	Femoral	6.21	1.33	4.88
Rat #212	45/55 Glucose/Fructose	Femoral	6.35	1.48	4.87
Rat #216	45/55 Glucose/Fructose	Femoral	5.32	1.10	4.22
Rat #219	45/55 Glucose/Fructose	Femoral	5.70	1.60	4.10
Rat #223	45/55 Glucose/Fructose	Femoral	6.63	1.83	4.80
Rat #226	45/55 Glucose/Fructose	Femoral	6.69	1.66	5.04
Rat #230	45/55 Glucose/Fructose	Femoral	7.19	1.63	5.56
Rat #233	45/55 Glucose/Fructose	Femoral	6.41	1.19	5.23
Rat #239	45/55 Glucose/Fructose	Femoral	8.13	2.00	6.13
Rat #102	Water	Portal	1.88	1.45	0.43
Rat #105	Water	Portal	2.01	1.73	0.27
Rat #108	Water	Portal	2.02	1.59	0.43
Rat #112	Water	Portal	1.91	1.80	0.11
Rat #114	Water	Portal	1.72	1.62	0.10
Rat #117	Water	Portal	2.19	1.87	0.32
Rat #120	Water	Portal	1.95	1.76	0.19
Rat #123	Water	Portal	1.79	1.72	0.07
Rat #204	Sucrose	Portal	51.34	0.86	50.48
Rat #205	Sucrose	Portal	26.04	1.15	24.89
Rat #211	Sucrose	Portal	42.39	1.23	41.15
Rat #221	Sucrose	Portal	38.59	1.67	36.92
Rat #222	Sucrose	Portal	34.32	1.66	32.66
Rat #228	Sucrose	Portal	27.38	1.76	25.62
Rat #231	Sucrose	Portal	23.40	1.30	22.10
Rat #236	Sucrose	Portal	32.66	1.22	31.44
Rat #237	Sucrose	Portal	37.04	1.77	35.28
Rat #101	Glucose	Portal	1.90	1.54	0.35
Rat #103	Glucose	Portal	1.67	1.43	0.24
Rat #107	Glucose	Portal	1.99	1.54	0.45
Rat #110	Glucose	Portal	1.90	1.74	0.16
Rat #115	Glucose	Portal	1.93	1.85	0.09
Rat #118	Glucose	Portal	2.38	1.74	0.64
Rat #122	Glucose	Portal	1.91	1.65	0.26
Rat #124	Glucose	Portal	1.99	1.81	0.18

S3 Table. Data for Fructose AdjC$_{max}$ in Femoral and Portal Veins.

Rat ID	Gavage Treatment	Vein	Fructose C_{max} (mg/dL)	Fructose Baseline (Time = 0) (mg/dL)	Fructose AdjC$_{max}$ (mg/dL)
Rat #104	Fructose	Portal	40.59	1.58	39.02
Rat #106	Fructose	Portal	39.19	2.55	36.63
Rat #109	Fructose	Portal	38.46	1.74	36.73
Rat #111	Fructose	Portal	60.21	1.73	58.48
Rat #113	Fructose	Portal	59.19	1.47	57.72
Rat #116	Fructose	Portal	37.50	1.81	35.69
Rat #119	Fructose	Portal	38.62	1.80	36.83
Rat #121	Fructose	Portal	29.61	1.60	28.01
Rat #201	45/55 Glucose/Fructose	Portal	35.35	0.85	34.51
Rat #203	45/55 Glucose/Fructose	Portal	50.91	0.85	50.06
Rat #212	45/55 Glucose/Fructose	Portal	37.76	1.17	36.59
Rat #216	45/55 Glucose/Fructose	Portal	24.20	0.68	23.52
Rat #219	45/55 Glucose/Fructose	Portal	28.00	1.75	26.24
Rat #223	45/55 Glucose/Fructose	Portal	31.71	1.81	29.90
Rat #226	45/55 Glucose/Fructose	Portal	43.78	1.64	42.13
Rat #230	45/55 Glucose/Fructose	Portal	35.17	1.32	33.85
Rat #233	45/55 Glucose/Fructose	Portal	30.35	1.05	29.30
Rat #239	45/55 Glucose/Fructose	Portal	41.17	1.91	39.26

AdjC$_{max}$ = maximum observed concentration - concentration at time = 0. C_{max} = maximum observed concentration.
