# Supplementary material for: Effects of fructose-containing sweeteners on fructose intestinal, hepatic, and oral bioavailability in dual-catheterized rats
Source: PLoS One. 2018 Nov 8;13(11):e0207024. doi: 10.1371/journal.pone.0207024 (PMC6224110; doi:10.1371/journal.pone.0207024)
Supplement: S4 Table — AdjCmax/D = adjusted maximum observed concentration normalized by fructose dose. AUC/D = area under the curve normalized by fructose dose. BW = body weight at sacrifice. D = fructose amount/body weight. Eh = hepatic extraction ratio. F = systemic bioavailability. Fa x Fg = intestinal availability. Fh = hepatic availability. Kb/p = whole blood to plasma concentration ratio. PV: portal vein. Qpv = portal blood flow. SYS: femoral vein. (PDF) [file pone.0207024.s004.pdf]

**S4 Table. Data for Fructose AdjC$_{max}$/D, AUC/D, and Bioavailabilty.**

| Rat ID | Gavage Treatment | BW (g) | Fructose Amount (mg) | Dose (mg/g) | Fructose AdjC$_{max_sys}$/D (mg/dL/(mg/g)) | Fructose AdjC$_{max_pv}$ (mg/dL/(mg/g)) | Fructose AUC$_{sys}$/D (mg/dl*min/(mg/g)) | Fructose AUC$_{pv}$/D (mg/dl*min/(mg/g)) | $Q_{pv}$ (ml/min/kg) | $K_{b/p}$ | $F_a$ x $F_g$ | $F_h$ | F | $E_h$ |
|---|---|---|---|---|---|---|---|---|---|---|---|---|---|---|
| Rat #104 | Fructose | 270.7 | 250 | 0.924 | 5.39 | 42.25 | 1104.53 | 3904.65 | 32.9 | 0.711 | 0.655 | 0.283 | 0.185 | 0.717 |
| Rat #106 | Fructose | 263.4 | 250 | 0.949 | 7.24 | 38.60 | 1277.88 | 3318.92 | 32.9 | 0.711 | 0.477 | 0.385 | 0.184 | 0.615 |
| Rat #109 | Fructose | 264.3 | 250 | 0.946 | 7.14 | 38.83 | 1192.80 | 3192.25 | 32.9 | 0.711 | 0.468 | 0.374 | 0.175 | 0.626 |
| Rat #111 | Fructose | 246.6 | 250 | 1.014 | 6.06 | 57.68 | 1198.40 | 5807.84 | 32.9 | 0.711 | 1.078 | 0.206 | 0.222 | 0.794 |
| Rat #113 | Fructose | 247.0 | 250 | 1.012 | 4.72 | 57.02 | 1109.42 | 4359.89 | 32.9 | 0.711 | 0.760 | 0.254 | 0.193 | 0.746 |
| Rat #116 | Fructose | 280.8 | 250 | 0.890 | 7.26 | 40.09 | 1242.32 | 2597.60 | 32.9 | 0.711 | 0.317 | 0.478 | 0.152 | 0.522 |
| Rat #119 | Fructose | 255.6 | 250 | 0.978 | 5.35 | 37.65 | 1199.97 | 3513.77 | 32.9 | 0.711 | 0.541 | 0.342 | 0.185 | 0.658 |
| Rat #121 | Fructose | 282.0 | 250 | 0.887 | 5.59 | 31.59 | 1275.26 | 4006.66 | 32.9 | 0.711 | 0.639 | 0.318 | 0.203 | 0.682 |
| Rat #201 | 45/55 Glucose/Fructose | 303.1 | 137.5 | 0.454 | 8.60 | 76.06 | 1601.73 | 4068.41 | 32.9 | 0.711 | 0.577 | 0.394 | 0.227 | 0.606 |
| Rat #203 | 45/55 Glucose/Fructose | 269.7 | 137.5 | 0.510 | 9.56 | 98.19 | 1639.92 | 4561.91 | 32.9 | 0.711 | 0.684 | 0.359 | 0.246 | 0.641 |
| Rat #212 | 45/55 Glucose/Fructose | 250.8 | 137.5 | 0.548 | 8.88 | 66.75 | 1411.67 | 3589.09 | 32.9 | 0.711 | 0.509 | 0.393 | 0.200 | 0.607 |
| Rat #216 | 45/55 Glucose/Fructose | 259.3 | 137.5 | 0.530 | 7.96 | 44.35 | 1872.04 | 4309.49 | 32.9 | 0.711 | 0.570 | 0.434 | 0.248 | 0.566 |
| Rat #219 | 45/55 Glucose/Fructose | 269.3 | 137.5 | 0.511 | 8.02 | 51.40 | 1779.96 | 3640.24 | 32.9 | 0.711 | 0.435 | 0.489 | 0.213 | 0.511 |
| Rat #223 | 45/55 Glucose/Fructose | 266.1 | 137.5 | 0.517 | 9.28 | 57.87 | 1907.86 | 4218.20 | 32.9 | 0.711 | 0.540 | 0.452 | 0.244 | 0.548 |
| Rat #226 | 45/55 Glucose/Fructose | 266.9 | 137.5 | 0.515 | 9.78 | 81.78 | 1753.55 | 4265.97 | 32.9 | 0.711 | 0.588 | 0.411 | 0.242 | 0.589 |
| Rat #230 | 45/55 Glucose/Fructose | 258.3 | 137.5 | 0.532 | 10.44 | 63.59 | 1941.10 | 3936.39 | 32.9 | 0.711 | 0.467 | 0.493 | 0.230 | 0.507 |
| Rat #233 | 45/55 Glucose/Fructose | 291.4 | 137.5 | 0.472 | 11.08 | 62.09 | 2050.05 | 3978.47 | 32.9 | 0.711 | 0.451 | 0.515 | 0.232 | 0.485 |
| Rat #239 | 45/55 Glucose/Fructose | 267.2 | 137.5 | 0.515 | 11.91 | 76.30 | 2454.68 | 4350.27 | 32.9 | 0.711 | 0.443 | 0.564 | 0.250 | 0.436 |
| Rat #204 | Sucrose | 282.8 | 125 | 0.442 | 7.05 | 114.21 | 1529.56 | 4296.58 | 32.9 | 0.711 | 0.647 | 0.356 | 0.230 | 0.644 |
| Rat #205 | Sucrose | 271.9 | 125 | 0.460 | 10.14 | 54.14 | 1652.44 | 3181.47 | 32.9 | 0.711 | 0.358 | 0.519 | 0.186 | 0.481 |
| Rat #211 | Sucrose | 261.3 | 125 | 0.478 | 6.28 | 86.02 | 1374.05 | 5530.11 | 32.9 | 0.711 | 0.972 | 0.248 | 0.242 | 0.752 |
| Rat #221 | Sucrose | 272.0 | 125 | 0.460 | 10.99 | 80.34 | 2232.43 | 4835.20 | 32.9 | 0.711 | 0.609 | 0.462 | 0.281 | 0.538 |
| Rat #222 | Sucrose | 272.0 | 125 | 0.460 | 7.98 | 71.07 | 2314.02 | 4484.45 | 32.9 | 0.711 | 0.508 | 0.516 | 0.262 | 0.484 |
| Rat #228 | Sucrose | 283.5 | 125 | 0.441 | 6.14 | 58.10 | 1920.06 | 4669.96 | 32.9 | 0.711 | 0.643 | 0.411 | 0.264 | 0.589 |
| Rat #231 | Sucrose | 268.1 | 125 | 0.466 | 8.93 | 47.39 | 1959.17 | 3974.39 | 32.9 | 0.711 | 0.471 | 0.493 | 0.232 | 0.507 |
| Rat #236 | Sucrose | 288.3 | 125 | 0.434 | 9.59 | 72.51 | 1927.81 | 3743.17 | 32.9 | 0.711 | 0.425 | 0.515 | 0.219 | 0.485 |
| Rat #237 | Sucrose | 280.4 | 125 | 0.446 | 11.45 | 79.14 | 2192.52 | 4104.75 | 32.9 | 0.711 | 0.447 | 0.534 | 0.239 | 0.466 |

AdjC$_{max}$/D = adjusted maximum observed concentration normalized by fructose dose. AUC/D = area under the curve normalized by fructose dose. BW = body weight at sacrifice. D = fructose amount/body weight. $E_h$ = hepatic extraction ratio.
F = systemic bioavailability. $F_a$ x $F_g$ = intestinal availability. $F_h$ = hepatic availability. $K_{b/p}$ = whole blood to plasma concentration ratio. PV: portal vein. $Q_{pv}$ = portal blood flow. SYS: femoral vein.
