# Supplementary material for: Effects of fructose-containing sweeteners on fructose intestinal, hepatic, and oral bioavailability in dual-catheterized rats
Source: PLoS One. 2018 Nov 8;13(11):e0207024. doi: 10.1371/journal.pone.0207024 (PMC6224110; doi:10.1371/journal.pone.0207024)
Supplement: S5 Table — CRBC = concentration in red blood cells. CPL = concentration in plasma. Kb/p = whole blood to plasma concentration ratio. (PDF) [file pone.0207024.s005.pdf]

S5 Table. Data for Whole Blood to Plasma Ratio of Fructose.

Compound	Sample#	Hematocrit (%)	C_{RBC} (μM)	C_{PL} (μM)	$K_{b/p}$
Fructose	1	43.5	255.3	637.8	0.739
	2	46.5	189.2	572.5	0.689
	3	43	179.0	573.9	0.704

C_{RBC} = concentration in red blood cells. C_{PL} = concentration in plasma. $K_{b/p}$ = whole blood to plasma concentration ratio.
